# Artificial Intelligence in Pulmonary Endoscopy: Current Evidence, Limitations, and Future Directions

**DOI:** 10.3390/jimaging12040167

**Published:** 2026-04-12

**Authors:** Sara Lopes, Miguel Mascarenhas, João Fonseca, Adelino F. Leite-Moreira

**Affiliations:** 1 Thoracic Surgery, Portuguese Institute of Oncology of Porto, 4200-072 Porto, Portugal; 2 Faculty of Medicine, University of Porto, 4200-437 Porto, Portugal; 3 Precision Medicine Unit, Department of Gastroenterology, Hospital São João, 4200-437 Porto, Portugal; 4 World Gastroenterology Organisation Training Center, 4200-437 Porto, Portugal; 5 Department of Cardiothoracic Surgery, Hospital São João, 4200-437 Porto, Portugal

**Keywords:** artificial intelligence, bronchoscopy, endobronchial ultrasound, deep learning, pulmonary endoscopy, computer-aided detection, computer-aided diagnosis

## Abstract

Background: Artificial intelligence (AI) is increasingly applied in pulmonary endoscopy, including diagnostic bronchoscopy, interventional pulmonology and endobronchial imaging. Advances in computer vision, machine learning and robotic systems have expanded the potential for automated lesion detection, navigation to peripheral pulmonary lesions, and real-time procedural support. However, the current evidence base remains heterogeneous, and translational challenges persist. Methods: This review summarizes current applications and developments of AI across white-light bronchoscopy (WLB), image-enhanced bronchoscopy (e.g., narrow-band imaging and autofluorescence imaging), endobronchial ultrasound (EBUS), virtual and robotic bronchoscopies, and workflow optimization and training. The authors also examine the methodological limitations, regulatory considerations, and implementation barriers that affect translation into routine practice. Results: Reported developments include deep learning-based models for mucosal abnormality detection, lymph-node characterization during EBUS-guided transbronchial needle aspiration (EBUS-TBNA), improved lesion localization, and reduction in operator-dependent variability. Additionally, AI-assisted simulation platforms and decision-support tools are reshaping training paradigms. Nevertheless, most studies remain retrospective or single-center, with limited external validation, dataset heterogeneity, unclear model explainability, and incomplete integration into clinical workflows. Conclusions: AI has the potential to support lesion detection, navigation, and training in pulmonary endoscopy. However, robust prospective validation, standardized datasets, transparent model reporting, robust data governance, multidisciplinary collaboration, and careful integration into clinical practice are required before widespread adoption.

## 1. Introduction

Pulmonary endoscopy is central to the diagnosis and management of a wide range of thoracic diseases, including lung cancer (LC), interstitial lung disease (ILD) and airway disorders. Over the past decade, bronchoscopic practice has expanded beyond conventional white-light inspection, to include image-enhanced bronchoscopy, EBUS, navigation systems, and advanced tissue acquisition techniques [1,2]. EBUS, particularly convex probe (CP-) EBUS-TBNA, has transformed mediastinal and hilar staging in LC, providing minimally invasive access to lymph-node (LN) and mediastinal lesions, with high sensitivity and specificity [3,4,5]. Beyond mediastinal staging, EBUS-based cryobiopsy, and transbronchial mediastinal cryobiopsy, are emerging to obtain larger tissue samples for complex malignant and benign conditions [6]. Transbronchial lung cryobiopsy (TBLC), via flexible bronchoscopy, is increasingly incorporated into diagnostic algorithms for fibrotic ILD, offering larger and better-preserved specimens than conventional forceps biopsy, with growing evidence on diagnostic yield and safety, when performed in experienced centers [7,8,9,10]. In parallel, interventional pulmonology has evolved into a dedicated subspecialty for more technically demanding procedures, encompassing rigid bronchoscopy, advanced airway and pleural interventions, and complex sedation and anesthesia protocols [1,11]. These developments have improved access to peripheral and mediastinal pathology, but also increased procedural and interpretative complexity.

At the same time, thoracic imaging and endoscopic decision-making have become markedly more complex. High-resolution computed tomography (HRCT), tomography positron emission scan (PET-CT), and multimodal endobronchial imaging (white-light, autofluorescence, narrow-band imaging, radial and convex EBUS) are able to generate large volumes of heterogeneous data that must be integrated with clinical, functional, and molecular information [3,4,5,6,12]. Multiple bronchoscopic platforms (e.g., radial EBUS, electromagnetic and virtual bronchoscopy navigation, and more recently robotic bronchoscopy) have expanded diagnostic reach for peripheral lung lesions, with the introduction of a wider range of procedural options and technical parameters [2,3]. Within mediastinal evaluation, several factors influence sampling strategy and interpretation (lymph-node size, PET uptake, EBUS sonographic features, elastography patterns, and prior therapies) [3,4,5,6,12]. Decision-making during bronchoscopy and EBUS therefore requires rapid interpretation of multimodal data in time-critical settings, with important implications for diagnostic yield, staging accuracy, and procedural safety. Variability in operator expertise, image interpretation and procedural strategy remains a recognized limitation, particularly in technically demanding procedures, such as peripheral lesion sampling and mediastinal staging.

**Figure 1:**
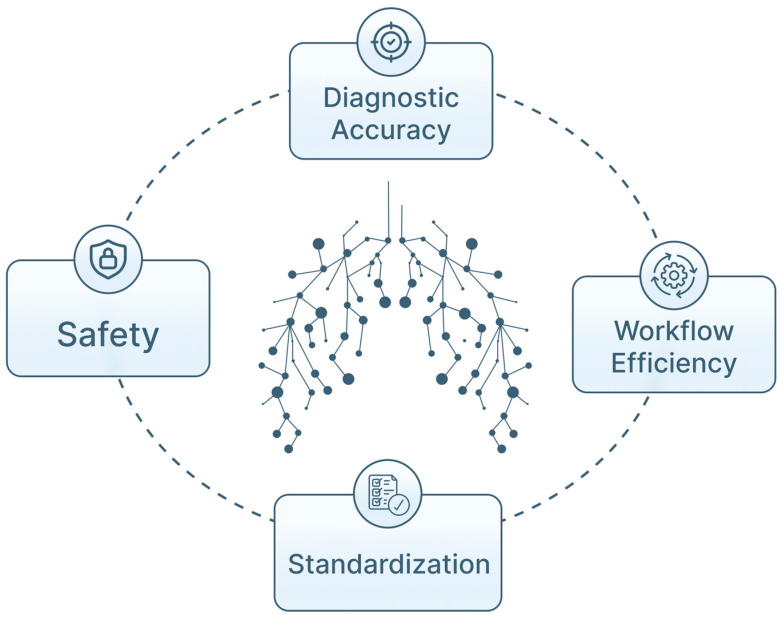
Conceptual overview of the potential clinical impact of AI in pulmonary endoscopy. AI-assisted systems may contribute to improved diagnostic accuracy, enhanced workflow efficiency, procedural safety, and greater standardization of bronchoscopic, and endobronchial ultrasound practices, while remaining integrated within clinician decision-making.

The rationale for applying AI in pulmonary endoscopy is multifaceted (Figure 1). Machine learning (ML) and deep learning (DL) approaches are increasingly used to support image interpretation, navigation and procedural decision-making in this data-rich environment [11,13]. In other image-intensive specialties, AI has improved detection performance, interobserver variability and optimization of workflows. Similar applications are now being investigated in bronchoscopy and EBUS for lesion detection, LN characterization, segmentation, frame selection, navigation support and risk stratification of peripheral nodules, with potential to improve mediastinal staging accuracy [12,13,14,15,16,17,18,19,20]. Beyond image analysis, AI is also explored for procedural planning, structured reporting, quality metrics, and training [4,9,13,21]. These applications may support standardization across centers and operators, contributing to more consistent bronchoscopic and EBUS practice, while maintaining clinician oversight [4,5,13,16].

Explainable and trustworthy AI approaches emphasize transparency, robustness and bias mitigation. These principles are of utmost importance for clinician trust, safe use in high-risk procedural settings, and directly relevant to bronchoscopy and EBUS, where AI models are being developed to assist with lesion detection, characterization, and navigation [21,22,23]. However, most pulmonology studies remain retrospective or single-center, with heterogeneous datasets, limited external validation and unresolved challenges related to workflow integration, and regulatory oversight [16,17,18,19,20]. This review summarizes contemporary applications of AI in pulmonary endoscopy, evaluates the maturity and limitations of the existing evidence, and discusses clinical, methodological and regulatory challenges that must be addressed, in order to enable safe and effective implementation. By integrating evidence from recent studies, and AI in the medical literature, this review seeks to delineate where AI is already adding value in pulmonary endoscopy, identifying gaps that need to be addressed before routine adoption, and outlining priorities for future research and collaborative development.

## 2. Methods

This review was conducted as a narrative synthesis of the current literature on AI applications in pulmonary endoscopy, including bronchoscopy, EBUS, navigational techniques, and interventional pulmonology. A structured literature search was performed in PubMed/MEDLINE and Scopus, to identify relevant studies published up to January 2026. The search strategy combined terms related to AI, (“artificial intelligence”, “machine learning”, “deep learning”, “computer-aided detection”, “computer-aided diagnosis”), with pulmonary endoscopy concepts (“bronchoscopy”, “EBUS”, “pulmonary endoscopy”, “interventional pulmonology”). Boolean operators (AND/OR) were used to refine the search. Additional relevant articles were identified through manual screening of reference lists and recent review articles.

Eligible publications included original research articles, clinical studies (prospective and retrospective), technical validation studies, and relevant methodological or regulatory papers addressing AI applications in bronchoscopic imaging, EBUS, navigation, procedural decision support, or quality assessment. Studies unrelated to pulmonary endoscopy, purely theoretical AI research without clinical application, and non-English publications were excluded. Although this paper was not designed as a systematic review, efforts were made to enhance transparency and reduce selection bias. The review process was structured in accordance with the principles of the SANRA framework, including clearly defined search strategy, study selection criteria, and critical appraisal of the literature.

Given the heterogeneity of study designs, imaging modalities and reported outcome, a quantitative meta-analysis was not feasible. Instead, findings were synthezised qualitatively, with emphasis on clinical relevance, methodological robustness, evidence maturity and translational potential. To improve comparability across studies, key methodological variables were systematically extracted, when available, including study design (prospective vs. retrospective), setting (single-center vs. multi-center), dataset size, class imbalance, and validation strategy (internal vs. external, offline vs. real-time). Particular attention was given to data partitioning approaches (e.g., patient-level vs. frame-level splits), as frame-level partitioning may introduce data leakage and overestimate model performance.

Evidence maturity was appraised based on study design and validation characteristics, rather than reported performance metrics alone, and categorized into technical feasibility, retrospective clinical validation, prospective or real-time evaluation, and evidence synthesis. Finally, the review incorporates considerations from current regulatory and ethical frameworks relevant to AI-based medical devices, including requirements for transparency, validation, and safe clinical integration.

## 3. Foundations and Clinical Applications of AI in Pulmonary Endoscopy

AI in pulmonary endoscopy encompasses a range of computational methods designed to extract clinically meaningful information from endoscopic imaging, ultrasound, and multimodal diagnostic data. Understanding the conceptual foundations of these systems is essential to contextualize their current and emerging applications in bronchoscopy, EBUS, cryobiopsy, and interventional pulmonology.

### 3.1. AI for Image Enhancement and Multimodal Fusion

Advances in AI have enabled significant progress in image enhancement and multimodal data integration in pulmonary endoscopy. Pulmonary endoscopy is performed under challenging visual conditions, including low illumination, motion artifacts, variable tissue appearance, and restricted fields of view. In addition, bronchoscopic decision-making often requires integration of heterogeneous data sources, such as endoscopic video, ultrasound imaging and pre-procedural computed tomography. These factors contribute to variability in lesion detection, navigation accuracy and procedural strategy.

**Figure 2:**
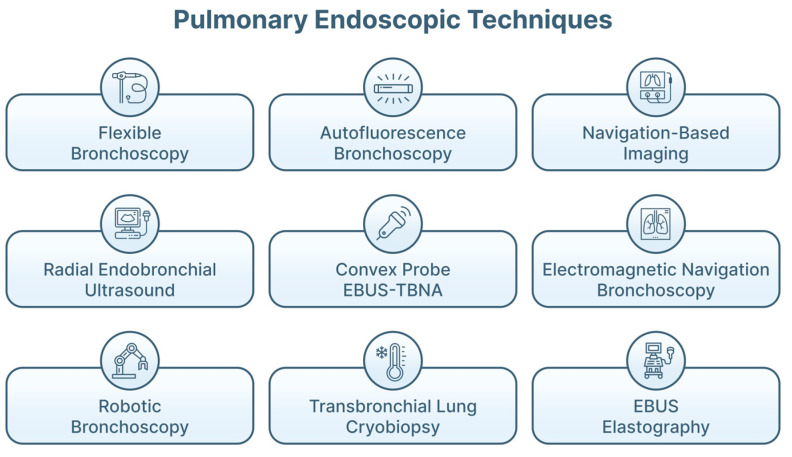
Pulmonary endoscopic techniques. This figure shows the principal bronchoscopic modalities used in pulmonary diagnostics and intervention. White-light bronchoscopy (WLB) provides baseline endoluminal visualization, while advanced imaging techniques such as narrow-band imaging (NBI) and autofluorescence bronchoscopy (AFB) enhance mucosal contrast and facilitate early lesion detection. Radial endobronchial ultrasound (rEBUS) allows assessment of peripheral pulmonary lesions, and convex probe EBUS (CP-EBUS) enables real-time visualization and sampling of mediastinal and hilar lymph nodes. These techniques generate complementary visual and ultrasound data, which can be integrated with pre-procedural imaging to support AI-driven applications, including computer-aided detection (CADe), diagnosis (CADx), and navigation, within a multimodal clinical workflow.

AI-based image enhancement and multimodal fusion techniques aim to address these limitations. Image enhancement methods improve visual quality, whereas multimodal models align endoscopic views with pre-procedural imaging, enabling anatomical segmentation. Together, these approaches support lesion localization, navigation to peripheral targets, and intraprocedural decision-making. An overview of pulmonary endoscopic techniques and their clinical roles is provided in Table 1, while Figure 2 illustrates representative imaging modalities relevant to AI integration.

#### 3.1.1. Machine Learning vs. Deep Learning: Convolutional Neural Networks, Transformers, and 3D Architectures

**Figure 3:**
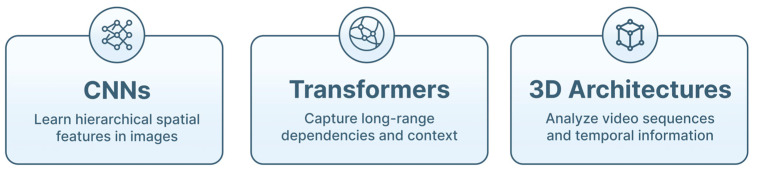
Dominant deep learning models for image-based medical artificial intelligence. This figure illustrates the main DL model types used in medical image analysis. These architectures underpin applications such as lesion detection, classification, segmentation, and procedural guidance in medical imaging.

ML encompasses algorithms that learn patterns from data to perform tasks such as classification, prediction or clustering. Traditional ML methods, including support vector machines and ensemble approaches, remain useful for structured clinical variables. However, the high dimensionality and visual complexity of endoscopic imaging have shifted most applications toward DL [24,25]. Convolutional neural networks (CNNs) excel at learning hierarchical spatial features, being the most widely used DL architecture in pulmonary endoscopy (Figure 3) [26,27]. CNN-based models have achieved high performance in tasks such as bronchoscopic lesion detection, LN segmentation on EBUS, and classification of radial EBUS (rEBUS) images [26,27,28]. Their ability to process pixel-level information without handcrafted feature engineering is a key advantage in complex visual environments like the airways [28,29]. Emerging transformer-based architectures, originally developed for natural language processing (NLP), have recently demonstrated strong performance in medical imaging due to their ability to model long-range dependencies and global image context [24,30]. Vision transformers (ViTs) and hybrid CNN–transformer models have been applied to thoracic CT and multimodal fusion tasks, setting state-of-the-art benchmarks in pulmonary nodule classification and segmentation [31]. Their adoption in endoscopic imaging is accelerating as datasets expand and computational efficiency improves. In addition, 3D DL architectures (e.g., 3D CNNs and spatiotemporal networks) enable the analysis of full video sequences rather than isolated frames. These models capture temporal motion information, which is particularly relevant in bronchoscopy and EBUS (where tissue deformation, probe movement, and respiratory motion influence image appearance) [31]. 3D networks have been used to automatically extract representative frames from CP-EBUS videos, perform video-based LN characterization, and support navigation in robotic bronchoscopy [26]. The principal AI architectures used in this field are summarized in Table 2.

**Super-Resolution and Denoising in Bronchoscopic Videos**

**Figure 4:**
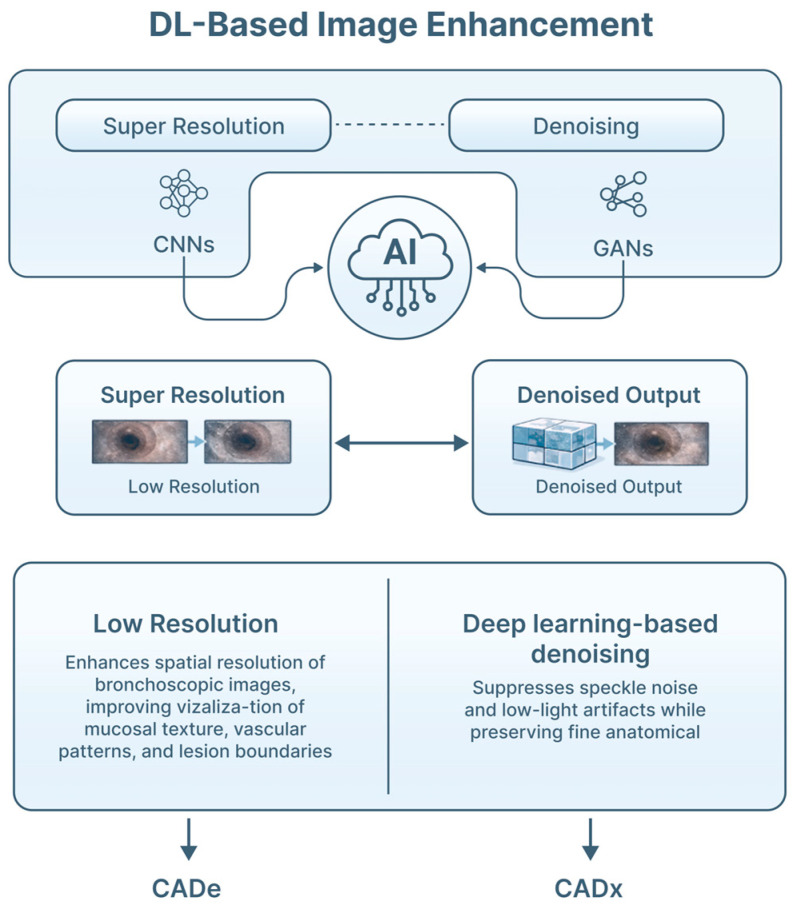
DL-based image enhancement techniques in bronchoscopic imaging. Super-resolution methods use DL models to enhance spatial resolution of bronchoscopic images, improving visualization of mucosal texture, vascular patterns, and lesion boundaries. Denoising approaches suppress speckle noise and low-light artifacts while preserving fine anatomical detail, avoiding distortion of subtle pathological features. These techniques are primarily implemented using convolutional neural networks (CNNs) and generative adversarial networks (GANs), which learn mappings between low- and high-resolution endoscopic image pairs. CADe—computed-aided detection; CADx—computer-aided diagnosis; DL—deep learning.

Bronchoscopic video quality is frequently degraded by limited spatial resolution, sensor noise, motion blur and compression artifacts, particularly in distal airways. These factors can obscure subtle mucosal abnormalities and reduce the performance of downstream computer-aided detection (CaDe) and diagnosis (CaDx) systems (Figure 4).

DL-based image enhancement methods, including super-resolution reconstruction and denoising, are increasingly investigated to address these issues. CNNs and generative adversarial networks (GANs) have shown the ability to reconstruct higher-resolution images from low-resolution inputs, improving visualization of mucosal texture, vascular patterns and lesion boundaries [28,29]. Denoising models, such as autoencoders and residual CNN architectures, can suppress noise and low-light artifacts while preserving anatomical detail [32,33]. These approaches are especially relevant in bronchoscopy, where aggressive noise filtering can otherwise distort subtle pathologic features. Improved image quality not only enhances human interpretation, but also increases the robustness and accuracy of AI-based CADe and CADx systems operating on bronchoscopic video streams.

**AI-Assisted Segmentation of Airways and Lesions**

Accurate segmentation of airway anatomy and endobronchial or peribronchial lesions underpins navigation, targeting and procedural planning in pulmonary endoscopy. Manual segmentation is time-consuming and prone to interobserver variability, particularly in distal or anatomically distorted airways. AI-based segmentation methods are therefore increasingly used to provide reproducible anatomical mapping and lesion localization, providing a foundation for computed-assisted decision support during pulmonary endoscopy [16].

**Figure 5:**
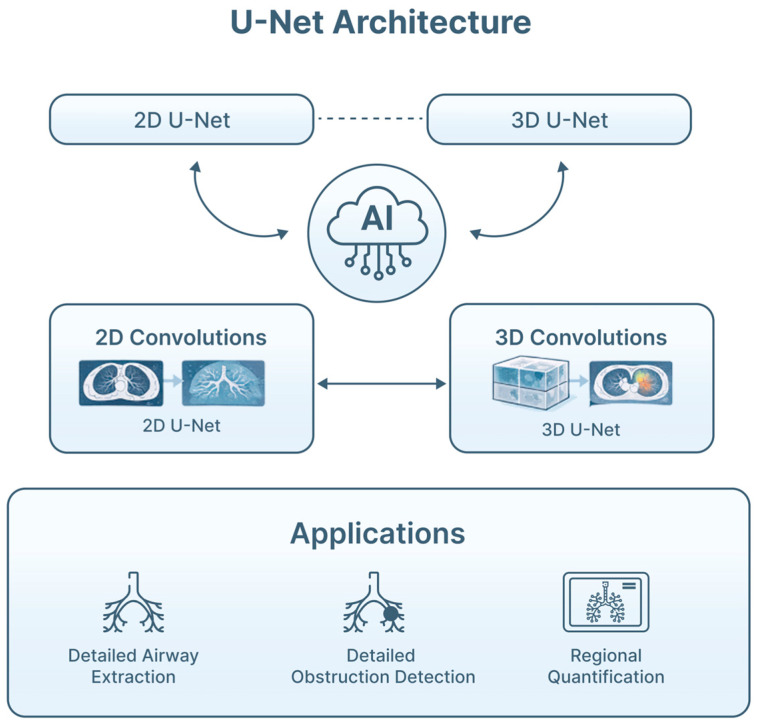
DL-based airway segmentation from CT imaging. U-Net-type architectures and 3D extensions enable reconstruction of airway anatomy, supporting navigation, image fusion and procedural planning. The U-Net architecture follows an encoder–decoder design, in which the encoder progressively extracts hierarchical features through downsampling, while the decoder reconstructs spatial detail through upsampling. Skip connections link corresponding encoder and decoder layers, preserving fine anatomical information critical for delineating airway structures. The 3D extension of U-Net applies volumetric convolutions to entire CT volumes, rather than individual slices, enabling the model to capture inter-slice spatial continuity and complex three-dimensional airway geometry. These properties make 3D U-Net architectures particularly well suited for accurate segmentation of central and peripheral airways, supporting applications such as navigational bronchoscopy, multimodal image fusion, and procedural planning in pulmonary endoscopy.

**Figure 6:**
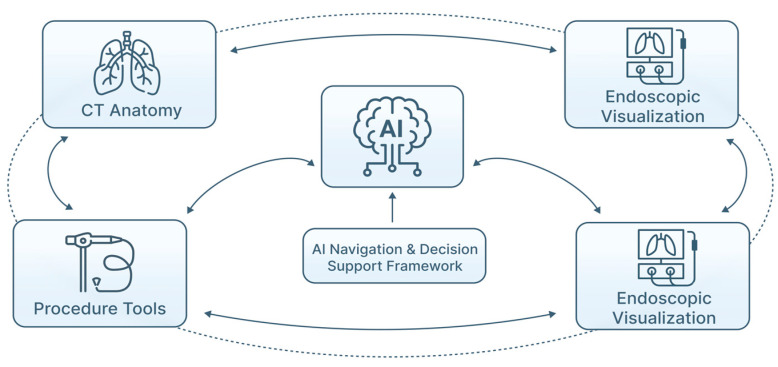
AI-assisted segmentation in multimodal fusion for pulmonary endoscopy. Segmentation algorithms delineate airway anatomy and relevant structures from pre-procedural CT, enabling accurate spatial models of the bronchial tree. These segmented anatomical maps are aligned with real-time bronchoscopic visualization, facilitating spatial registration and navigation during procedures. By linking CT anatomy, endoscopic imaging, and procedural tools within a unified framework, AI-assisted segmentation supports coherent navigation, real-time decision support, and improved procedural accuracy under clinician oversight.

DL approaches, especially U-Net-derived architectures and their 3D extensions, are widely applied for airway segmentation on thoracic CT (Figure 5). These systems can reconstruct bronchial trees to subsegmental levels, supporting virtual pathway planning to peripheral lesions [24,30]. Segmentation accuracy has improved significantly compared with traditional rule-based methods, particularly in challenging regions affected by partial volume effects or disease-related distortion. In EBUS, CNN models have been developed to segment LN and adjacent structures, facilitating downstream classification and biopsy guidance [16,26,31]. Similar strategies are being explored in bronchoscopic video analysis to delineate mucosal abnormalities and quantify lesion extent, supporting image-guided interventions. Collectively, AI-assisted segmentation plays a central role in multimodal fusion pipelines, linking CT anatomy, endoscopic visualization, and procedural tools into a coherent navigational and decision-support framework [16,30] (Figure 6).

#### 3.1.2. AI for Multimodal Fusion: Prediction and Prognostic Models of Malignancy Risk and Integration with Staging Algorithms (TNM)

**Figure 7:**
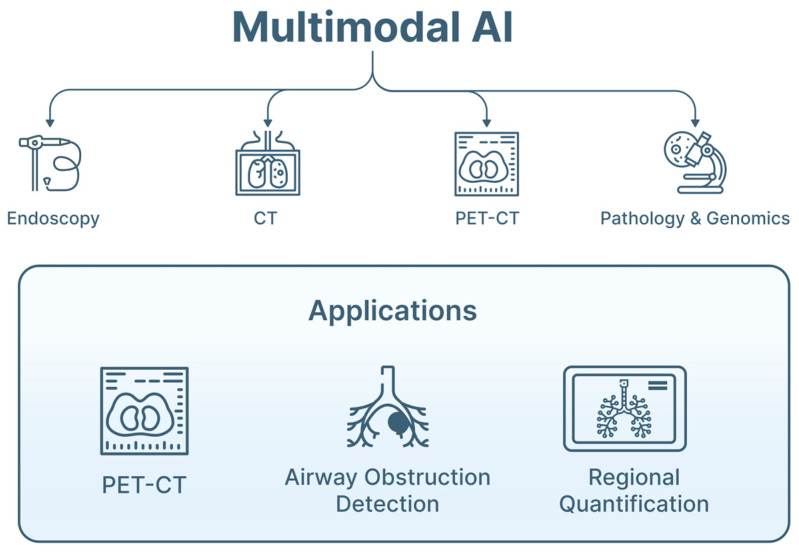
Conceptual framework of multimodal AI integration in pulmonary endoscopy. This figure illustrates a unified AI framework integrating multimodal data. Input data include bronchoscopic video streams, endobronchial ultrasound (EBUS) imaging (radial and convex probe), and pre-procedural imaging such as computed tomography (CT) and positron emission tomography (PET-CT), as well as optional clinical and pathological data. These heterogeneous inputs are processed through AI pipelines involving feature extraction, cross-modal alignment, and data fusion using ML and DL models. The framework reflects a human-in-the-loop paradigm, where AI augments but does not replace clinical judgment and highlights the potential for improved diagnostic accuracy, procedural efficiency, and standardization of care.

Pulmonary endoscopy increasingly operates within a multimodal diagnostic environment that combines endoscopic imaging, ultrasound, thoracic radiology and clinicopathological data. AI systems capable of integrating these complementary information sources are therefore being developed to support lesion characterization, staging and procedural planning (Table 3) [3,12,15,16,17,18,19,20]. Multimodal approaches have generally shown improved performance compared with single-modality models, particularly in oncological applications [25,28] (Figure 7).

For peripheral pulmonary lesions, integration of CT morphology, bronchoscopic findings and EBUS features has been explored to refine malignancy prediction and procedural targeting [17,18]. Radiological AI models provide complementary information, especially for subsolid or small nodules that may be difficult to characterize bronchoscopically. The addition of PET-derived metabolic data further enhances risk assessment in selected contexts, particularly for mediastinal LN evaluation [4,27].

In mediastinal evaluation, AI-assisted analysis of EBUS images combined with radiological or metabolic data can improve LN risk stratification beyond conventional sonographic criteria alone [4,16,17,18,19,20]. Such models estimate the probability of nodal malignancy, and may support more consistent selection of sampling sites, mainly in LC [4,16,17,18,19]. Similarly, fusion of CT-derived airway maps with real-time endoscopic imaging underpins AI-augmented navigation systems, particularly in robotic bronchoscopy (RB), where accurate alignment between pre-procedural imaging and intraprocedural anatomy is critical [26,28].

In the realm of tissue diagnosis, AI applied to digital pathology has achieved high performance in detecting malignancy, quantifying tumor microenvironment characteristics, and identifying actionable biomarkers [10,34]. Cryobiopsy samples provide larger, better-preserved architecture than forceps biopsy, making them particularly suitable for computational pathology approaches [8]. Genomic and transcriptomic data have begun to enter multimodal AI frameworks for LC and ILD. DL models incorporating radiology, pathology, and molecular features have shown promise for predicting oncogenic mutations, treatment response, and prognosis and demonstrated value in predicting survival and treatment response in LC [16,20,34,35].

**Figure 8:**
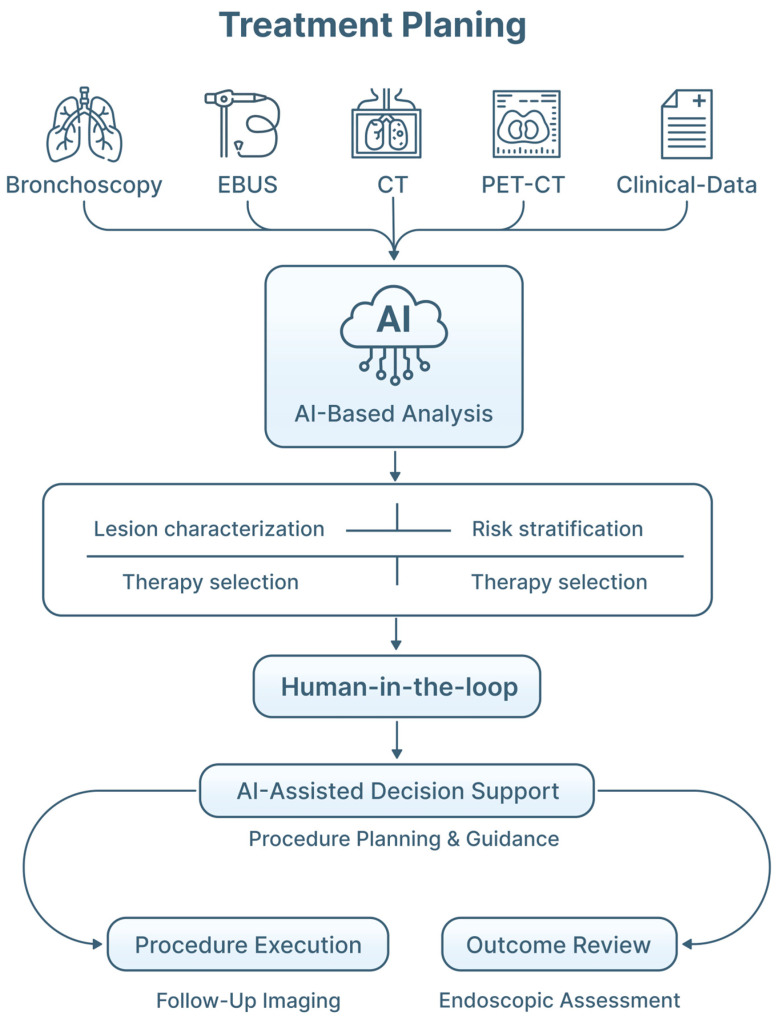
AI in treatment planning in pulmonary endoscopy. Multimodal diagnostic inputs (bronchoscopic imaging, EBUS, CT scan, PET-CT, and clinical data) are integrated by AI-based models to support lesion characterization, risk stratification, and selection of appropriate endobronchial therapeutic strategies. AI-assisted decision support informs procedural planning and guidance for bronchoscopic interventions, while post-treatment imaging and endoscopic assessment contribute to outcome evaluation and iterative refinement of treatment strategies. Throughout the workflow, AI functions as a clinical decision-support tool within a human-in-the-loop framework, preserving clinician oversight and responsibility.

**Figure 9:**
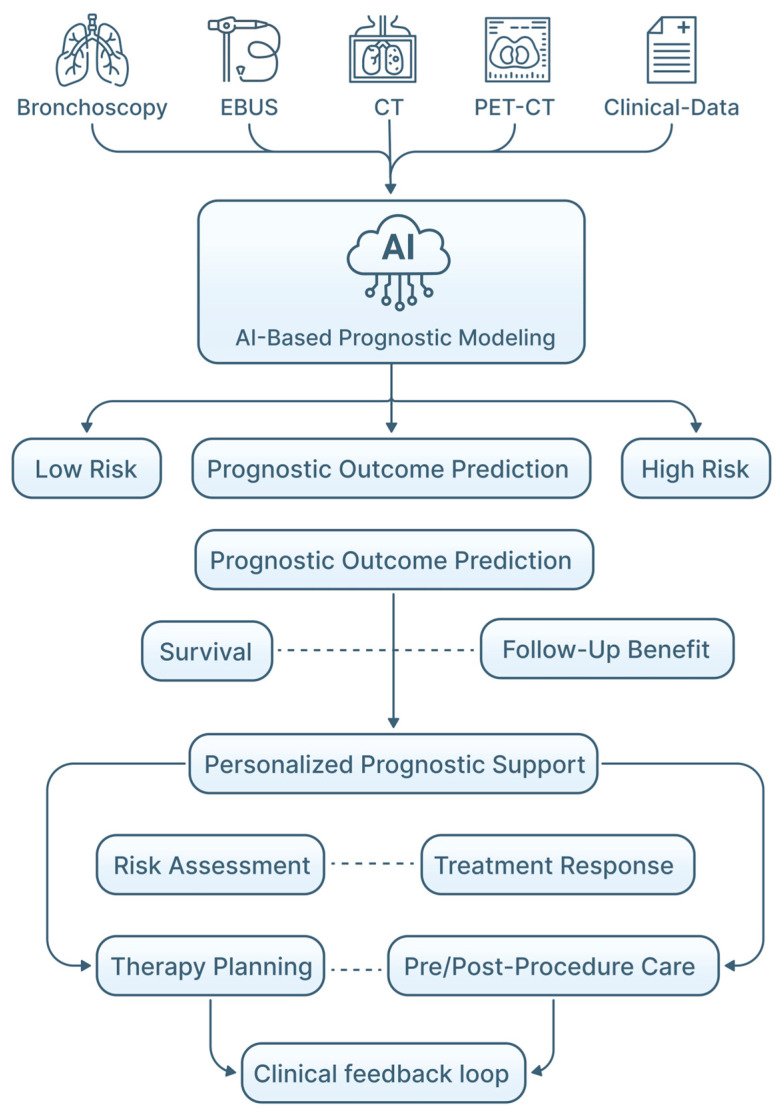
AI-assisted prognostic models in pulmonary endoscopy. Multimodal inputs, including bronchoscopic imaging, endobronchial ultrasound, radiologic data, and clinical information, are processed by AI algorithms to generate integrated prognostic assessments. AI-based models stratify patients into low-, medium-, and high-risk categories, and support prediction of clinically relevant outcomes such as survival and longitudinal follow-up results. These outputs inform personalized risk assessment, treatment planning, and post-procedural surveillance, while maintaining clinician oversight within a human-in-the-loop framework.

AI-driven predictive modeling also has potential implications for treatment planning in interventional pulmonology (Figure 8). AI systems capable of integrating bronchoscopic imaging with radiologic and histopathologic features may assist in identifying lesions suitable for endoscopic management, versus those requiring surgical or systemic treatment (Table 4). Although most AI-based treatment planning tools remain investigational, endobronchial therapies become more targeted and less invasive. AI-assisted prognostic models may help optimize patient selection, procedural timing, and therapeutic modality choice (Figure 9).

Predictive and prognostic AI models need prospective validation, transparency of model outputs, and alignment with established staging systems (TNM) before widespread clinical adoption. These tools will function as decision-support tools, rather than replacements for guideline-based algorithms [4,36].

### 3.2. AI for Disease Detection and Diagnosis

Early identification of endobronchial abnormalities is crucial for improving outcomes in LC, premalignant airway disease, and other mucosal pathologies. However, subtle lesions (e.g., carcinoma in situ, low-grade dysplasia) may be easily overlooked on standard WLB. AI offers new opportunities to improve detection accuracy by enhancing visual inspection, interpreting advanced imaging modalities, and providing real-time CADe during bronchoscopy.

#### 3.2.1. Computer-Aided Detection and Diagnosis Systems

Computer-aided detection (CADe) systems automatically highlight suspicious regions on medical images, functioning as alert tools that support visual inspection. Computer-aided diagnosis (CADx) extends this role by classifying detected findings and estimating the probability of malignancy or another pathology. CADe systems have improved lesion detection in other endoscopic fields, and similar approaches are now being explored in pulmonary endoscopy [37].

**Figure 10:**
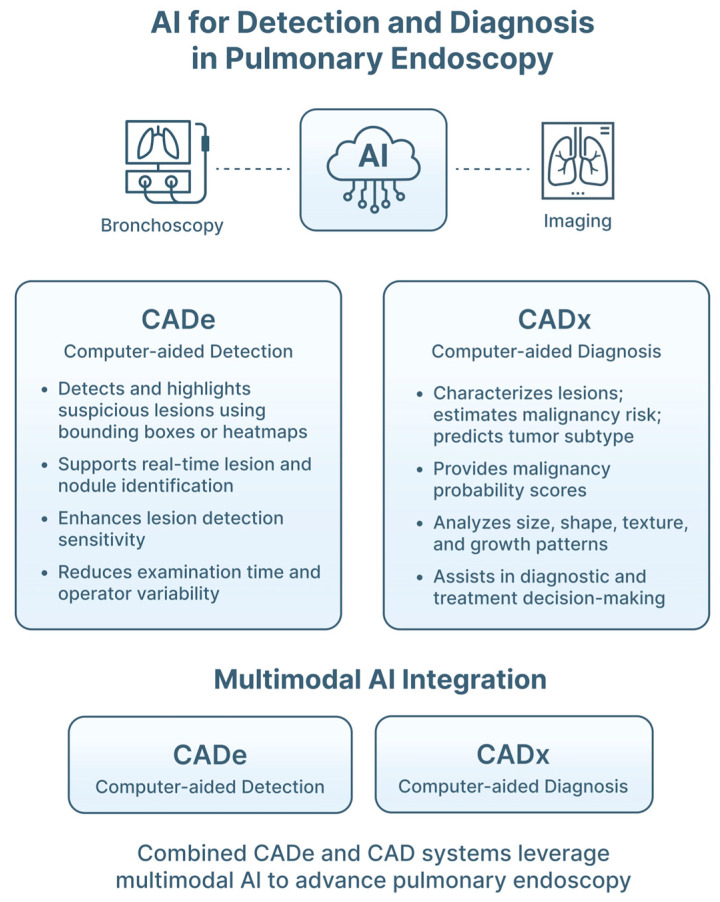
Computed-aided detection (CADe) and diagnosis (CADx) in pulmonary endoscopy. Input data include bronchoscopic video streams and endobronchial ultrasound (EBUS) imaging. AI models process data to perform CADe, identifying suspicious lesions or abnormal mucosal patterns, and CADx, providing classification of lesions (e.g., benign versus malignant) or lymph-node characterization. Outputs are delivered through real-time interfaces, such as visual overlays, heatmaps, and probability scores, to support targeted biopsy and clinical decision-making.

In bronchoscopy, CADe models have been developed to identify subtle endobronchial abnormalities, differentiate normal from abnormal mucosa, and identify sonographic LN features on EBUS imaging [26,38,39] (Figure 10). CADx systems aim to further characterize these findings, for example by classifying malignant versus benign pulmonary nodules, based on rEBUS images, predicting LN malignancy using features derived from CP-EBUS elastography and B-mode imaging, or estimating invasion and metastasis (Figure 10) [27,28,34]. CADx aims to support risk stratification and clinical decision-making, rather than merely highlighting abnormalities. An overview of reported CADe and CADx applications is provided in Table 5: reported performance metrics should be interpreted in the context of dataset size, class distribution, and validation strategy. As shown, most studies rely on retrospective, single-center datasets with internal validation only, and frequently lack reporting of class imbalance. This limits generalizability, and may overestimate model performance. External validation remains scarce, highlighting a key barrier to clinical translation.

**Deep Learning CADe Systems for White-Light Bronchoscopy: AI for Detection of Endobronchial Lesions**

Early airway neoplasia often presents as subtle mucosal irregularities that may be difficult to detect under WLB, even for experienced bronchoscopists [51]. Studies have shown that standard WLB has limited sensitivity for detecting high-grade dysplasia and early cancer [51]. DL CADe systems, typically based on CNNs, analyze bronchoscopic video frames in real time and highlight potential abnormalities using visual overlays. These systems aim to improve consistency of inspection, detection and outcomes, and reduce interobserver variability.

AI may support procedural quality by encouraging systematic airway inspection [16,32,52]. Temporal modeling approaches, including video-based architectures, have been explored to improve robustness to motion artifacts and variable lighting [44]. Early studies suggest that AI-assisted WLB can detect dysplasia, or early carcinoma, with high reported sensitivity and specificity, with area-under-the-curve (AUC) metrics exceeding 0.90 [16,32,52]. However, most data derive from retrospective single-center cohorts, and prospective validation across different bronchoscopic platforms remains limited [15,31]. Preliminary evidence also suggests that CADe systems can support trainees by standardizing lesion recognition and shortening learning curves [44,52,53].

**AI Integration with Light-Based Modalities: Autofluorescence, Narrow-Banding Imaging, and I-Scan**

Light-based imaging modalities provide enhanced contrast for detecting neoplastic mucosal changes, complementing WLB. AI is also being investigated in conjunction with these image-enhanced modalities to support standardized interpretation and reduction in interobserver variability. However, robust comparative studies are scarce.

Autofluorescence bronchoscopy (AFB) uses tissue autofluorescence to highlight dysplastic areas, offering high sensitivity, but limited specificity, due to confounding inflammation. ML models analyzing AFB have been explored to improve specificity, and have shown enhanced discrimination between inflammatory and dysplasia changes, compared with visual assessment alone [54,55].

Narrow-band imaging (NBI) enhances superficial microvascular patterns, early altered in neoplasia. In preliminary studies, AI models assessing microvascular patterns (e.g., tortuosity, dilation, irregular branching) have shown improved accuracy over expert bronchoscopists in early neoplasia detection [56].

Digital enhancement systems (e.g., i-scan) apply real-time post-processing algorithms to enhance mucosal texture and surface patterns. AI-assisted interpretation may help identify subtle mucosal lesions, although evidence remains limited [57]. Across all light-based modalities, AI can standardize interpretation, reduce interobserver variability, and generate objective quantifications of mucosal abnormalities, roles that are particularly valuable in early detection settings.

**AI in Virtual Bronchoscopy and Robotic Platforms**

Interventional bronchoscopy has become increasingly complex as clinicians navigate smaller, more peripheral, and anatomically challenging lesions. Precise localization of peripheral pulmonary lesions remains one of the most challenging aspects of interventional bronchoscopy. Even when navigation systems guide the bronchoscope to the correct airway generation, real-time confirmation of lesion proximity often requires rEBUS or fluoroscopy, both of which have limitations. rEBUS, electromagnetic navigation bronchoscopy (ENB), and robotic bronchoscopy (RB) diagnostic yields remain variable, particularly for lesions < 20 mm or without a bronchus sign. AI is emerging as a key enabler for improved navigation, real-time decision support, and enhanced procedural precision in these advanced bronchoscopic interventions. Virtual bronchoscopy (VB) and RB represent technological developments that intersect closely with AI-driven navigation and multimodal fusion.

**Figure 11:**
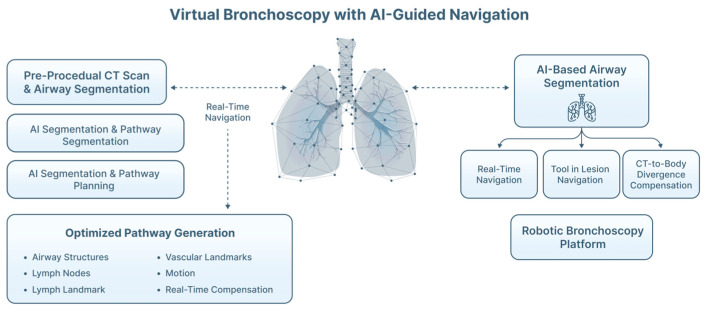
AI-guided navigation with virtual bronchoscopy (VB). AI has been incorporated into ENB platforms to refine dynamic navigation. ML algorithms can register live bronchoscopic video with preoperative CT-derived VB models, creating hybrid augmented-reality navigation environments.

Traditional VB systems rely on rule-based segmentation of airways, which can struggle with incomplete airway visibility, artifacts, or peripheral airway attenuation [58,59]. VB uses CT-derived three-dimensional airway reconstructions for pre-procedural planning and intra-procedural navigation. When integrated with CT-to-body registration models, AI-enhanced VB may partially compensate for anatomical deformation and respiratory motion, although divergence between pre-procedural imaging and intraprocedural anatomy remains a key limitation [59,60,61,62]. AI-based airway segmentation (particularly CNNs and 3D U-Net architectures) improves extraction of distal airways, supports pathway planning, and assists with automated target selection (Figure 11).

**Figure 12:**
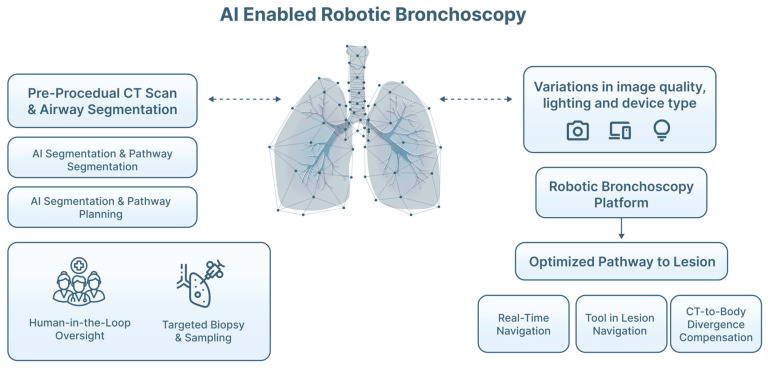
AI-enabled robotic bronchoscopy (RB). Pre-procedural thoracic computed tomography (CT) is processed using AI-based airway segmentation and pathway planning algorithms to generate an optimized route to peripheral pulmonary lesions. During the procedure, an RB platform provides articulated catheter control and enhanced distal stability, while AI-assisted navigation supports real-time lesion localization, tool-in-lesion confirmation, and compensation for CT-to-body divergence. The bronchoscopist remains in a human-in-the-loop role, integrating AI guidance with EBUS findings and clinical judgment to perform targeted sampling safely and effectively.

RB platforms have expanded reach into lung segments previously inaccessible by conventional bronchoscopy, providing enhanced catheter stability, distal reach and precise control compared with conventional techniques [16]. Current systems operate under clinician control, but increasingly incorporate AI-derived airway models, segmentation outputs and trajectory optimization [60,63,64]. These tools may improve the consistency of lesion access, particularly in small or anatomically challenging nodules [16,58,60,65] (Figure 12). Recent robotic platforms are increasingly leveraging AI for real-time trajectory prediction, airway deformation modeling, and optimal tool-path calculation [54]. Such systems can dynamically adjust guidance as the robotic catheter advances, potentially improving access to small or tortuous airways. Studies have demonstrated that AI algorithms can fuse robotic telemetry, bronchoscopic video, and rEBUS findings to refine lesion localization during RB procedures [63,64,65]. This multimodal data fusion supports automated repositioning suggestions, and it may eventually enable semi-autonomous robotic adjustments. Although most AI-enhanced robotic systems remain investigational, early clinical studies have shown that AI-guided RB improves navigational accuracy and may increase diagnostic yield, particularly for lesions < 2 cm or when the bronchus sign is absent [65].

However, neither VB nor RB independently resolve critical challenges such as CT-to-body divergence, or real-time lesion confirmation (Table 6). In practice, confirmation of target proximity still relies on adjunct modalities such as rEBUS or fluoroscopy [63,64,65,66]. AI-based multimodal fusion, integrating endoscopic video, ultrasound and CT-derived anatomy, is therefore central to the future evolution of these platforms [58,59].

Most available evidence relates to technical feasibility and navigation accuracy, rather than demonstrated improvement in clinical outcomes. VB and RB should thus be viewed as enabling platforms that may amplify the impact of AI-assisted segmentation, registration and decision support, rather than standalone solutions [63,64,65].

**AI in EBUS and Interventional Bronchoscopy**

**Figure 13:**
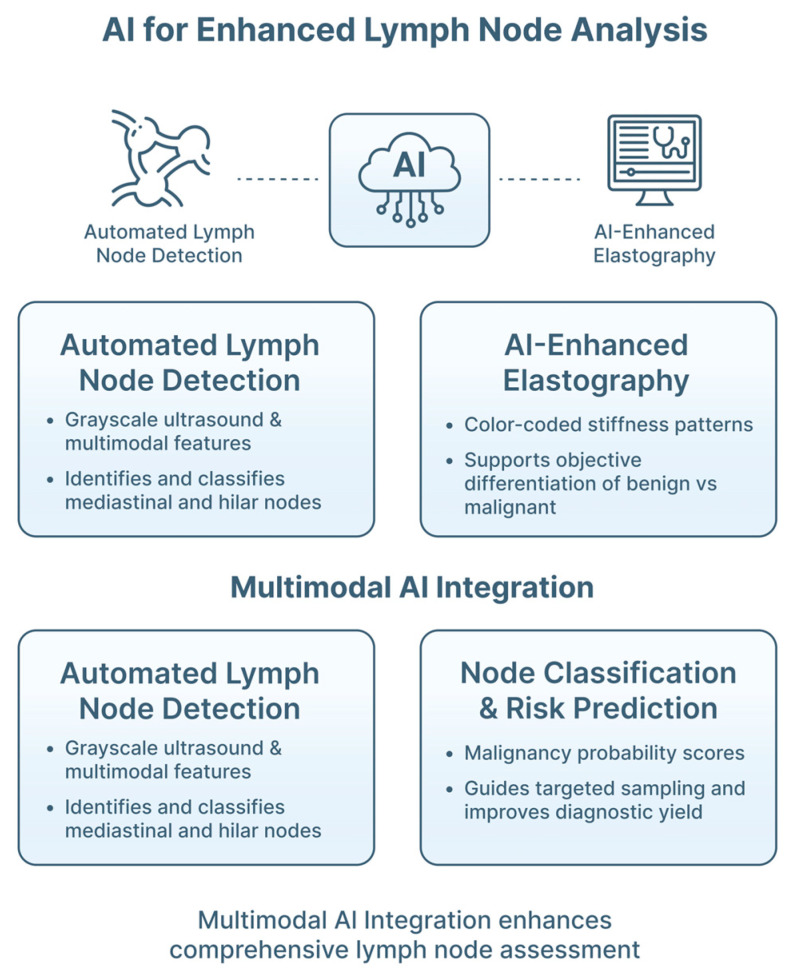
Current applications of artificial intelligence in EBUS. AI-based algorithms enable automated detection and classification of mediastinal and hilar LNs, using grayscale ultrasound and multimodal features. Integration of AI with EBUS elastography supports objective interpretation of tissue stiffness patterns, improving differentiation between benign and malignant nodes. AI-assisted systems contribute to improved diagnostic yield by optimizing lymph-node selection, reducing sampling errors, and standardizing nodal assessment. Emerging applications include AI-guided support for transbronchial needle aspiration (TBNA) and EBUS-guided cryobiopsy, facilitating precise targeting while maintaining clinician oversight within a human-in-the-loop framework.

EBUS has become an indispensable tool for mediastinal and hilar evaluation, staging of LC, and sampling of lymphadenopathy. Despite its central role, EBUS relies on multiple qualitative criteria: interpretation is highly operator-dependent, and diagnostic accuracy varies across centers and experience levels. Particularly DL-based image analysis has emerged as a promising avenue to enhance LN detection, segmentation and classification, improve elastography interpretation, and support real-time guidance during TBNA and cryobiopsy (Figure 13). DL reconstruction models can enhance interpretation and automated characterization of lesion–probe relationships, supporting decision-making regarding when to reposition the probe, adjust catheter angles, or reselect bronchial pathways [16].

(A) **Lymph-Node Detection and Classification**

Manual interpretation of CP-EBUS imaging requires integration of morphologic features (e.g., border distinctness, echogenicity, short-axis diameter, presence of necrosis, and hilar structures), which varies significantly among operators.

AI systems have been developed to automatically detect and segment LNs in EBUS imaging, reducing reliance on manual identification. DL models, particularly CNNs, have achieved high accuracy in identifying boundaries and differentiating LNs from adjacent vascular and mediastinal structures [16]. Ervik et al. demonstrated that CNN models can automatically select high-quality CP-EBUS frames, delineate LN boundaries on EBUS imaging, and differentiate nodes from adjacent structures, potentially improving TBNA accuracy and malignancy prediction [16].

Multimodal models incorporating B-mode, Doppler and elastography features have been investigated for LN classification, with performance in some studies comparable to expert interpretation [19,66,67]. Daneshpajooh et al. developed a DL classifier integrating texture, echo pattern, and vascularity metrics, achieving diagnostic performance comparable to expert bronchoscopists, and significantly improving reproducibility [66]. These tools help standardize interpretation, and may reduce the subjectivity associated with traditional qualitative criteria.

(B) **Elastography Interpretation**

EBUS elastography assesses tissue stiffness, with malignant nodes generally appearing stiffer than benign ones. Although elastography improves diagnostic accuracy, interpretation is highly dependent on operator experience, and interobserver variability remains substantial [19].

AI-enhanced elastography analysis has shown potential to standardize and enhance predictive accuracy, reducing subjectivity in stiffness pattern assessment. ML models quantifying color distribution patterns, strain ratios, and texture heterogeneity have demonstrated improved diagnostic discrimination over traditional visual scoring [19]. Izumo et al. highlighted that elastography patterns are associated with malignancy probability, but subjectivity limits consistency [19]. Subsequent AI-enhanced elastography studies, including automated color histogram and stiffness map analysis, have reported increased accuracy in distinguishing malignant from benign LNs, reducing interobserver variability, and supporting standardized staging [19].

(C) **Improving Diagnostic Yield and Reducing Sampling Errors**

Despite advances, false-negative rates in EBUS-TBNA remain influenced by inadequate sampling, inaccurate probe–lesion positioning, and variability in lesion visualization.

AI systems can support bronchoscopists in optimizing sampling locations and angles. AI-enhanced mapping of LN stations, and automated identification of vascular structures, may help prevent inadvertent sampling of non-target tissue or blood vessels, reducing procedural complications and increasing sensitivity for malignancy [16]. DL models capable of predicting malignancy likelihood for each LN subregion may eventually guide targeted sampling during real-time procedures. Real-time diagnostic-support algorithms may reduce sampling errors by flagging suboptimal probe positioning, or insufficient visualization before needle advancement [16,19]. Ebrahimian et al. demonstrated that a DL model could automatically select high-quality CP-EBUS frames, potentially improving TBNA accuracy by guiding needle placement toward optimal imaging views [16]. In peripheral lesion sampling, AI-assisted analysis of rEBUS and navigation data has been proposed to support lesion confirmation and targeting, particularly in small or technically challenging nodules. However, most systems have been evaluated in retrospective or simulated settings.

(D) **AI Potential in Guiding Criobiopsy: Interstitial Lung Disease**

With the purpose to reduce interobserver variability and support earlier and more standardized diagnosis, AI is increasingly being applied in the evaluation of ILD. DL models trained on high-resolution computed tomography (HRCT) have demonstrated the ability to classify ILD patterns, including usual interstitial pneumonia (UIP) and non-specific interstitial pneumonia (NSIP), with performance comparable to expert radiologists in selected settings.

Cryobiopsy samples provide larger, better-preserved architecture than forceps biopsy, making them particularly suitable for computational pathology approaches [8]. In mediastinal cryobiopsy, where tissue acquisition requires precise positioning of the cryoprobe relative to mediastinal structures, AI has the potential to support optimal probe placement, avoid vascular hazards, and ensure adequate sample size [6,16]. Given the growing role of cryobiopsy as a minimally invasive alternative to surgical lung biopsy in ILD diagnosis, AI tools are also being explored for the analysis of transbronchial lung cryobiopsy specimens. ML and DL algorithms can assist in identifying fibrosis patterns, quantifying architectural distortion, and detecting subtle histopathological features considered challenging to assess visually. Although applications of AI in EBUS-guided mediastinal cryobiopsy remain exploratory, early computational modeling using EBUS-derived imaging data demonstrates feasibility for AI-assisted planning of cryobiopsy trajectories [6]. As EBUS-guided transbronchial mediastinal cryobiopsy gains clinical traction, AI integration may play a central role in maximizing diagnostic yield while improving procedural safety, particularly in technically challenging stations such as 4L or 7.

Emerging multimodal approaches integrate radiological and histopathological data, combining CT-derived features with tissue-based analysis to improve diagnostic confidence and disease subtyping. Such models have the potential to reflect the multidisciplinary diagnostic process currently used in ILD evaluation.

While AI-assisted ILD assessment is promising, its integration into clinical workflows requires further standardization and robust external validation. Current evidence remains limited by heterogeneous datasets, variability in imaging acquisition and pathological annotation, and a lack of large-scale prospective validation.

#### 3.2.2. AI for Quality Assessment: Quality-Monitoring Tools

Bronchoscopic reporting is often heterogeneous, with variable terminology, incomplete documentation of inspected airway segments, and inconsistent reporting of EBUS findings. This variability hampers auditability, data reuse, and quality improvement initiatives [53,68].

Unlike gastrointestinal endoscopy, pulmonary endoscopy lacks widely standardized and routinely audited quality indicators [32,37,41,69]. Procedural completeness, airway inspection coverage, and systematic nodal assessment are often operator-dependent, contributing to variability in diagnostic yield and reproducibility [13,37]. AI-based quality-monitoring tools are therefore being explored to support more objective and auditable practice.

Current approaches focus on three main areas: assessment of airway inspection coverage, recognition of anatomical landmarks and procedural steps, and generation of automated quality proxies [68,70]. Using computer vision and DL, AI systems can analyze bronchoscopic video to identify bronchial segments, track visualization patterns, and detect potential blind spots [32,39]. Similar methods have been proposed in EBUS to support systematic LN station assessment and documentation of sampled versus unsampled nodes [13,35,37]. These tools aim to reduce missed lesions, support training, and facilitate continuous quality improvement. AI-generated quantitative metrics, such as segmental coverage, stability of visualization, and frame selection, have been proposed as proxies for procedural completeness [13,32,35,69,71]. Yoo et al. demonstrated that DL-based airway classification in bronchoscopy videos enables automatic identification of airway anatomy and procedural mapping [44]. Zhi et al. developed in 2021 an ML model to automatically select informative frames from EBUS strain videos, reducing subjective operator choice and enabling standardized downstream interpretation [42]. AI-based quality metrics have been proposed to evaluate systematic nodal assessment, confirm correct LN station sampling, and verify adherence to guideline-recommended staging algorithms [70]. However, these approaches remain largely exploratory, and there are currently no prospectively validated AI systems equivalent to established quality indicators used in other endoscopic fields. Evidence is primarily limited to feasibility and proof-of-concept studies, and consensus on clinically meaningful quality metrics is still evolving.

NLP and multimodal AI models are being investigated to extract procedural findings from video, ultrasound images and structured metadata, enabling more consistent and structured reporting [72]. Such systems may improve auditability, reduce reporting heterogeneity, and support large-scale data aggregation, aligning with broader efforts to standardize interventional pulmonology practice [68,70].

## 4. Technical and Methodological Considerations

**Figure 14:**
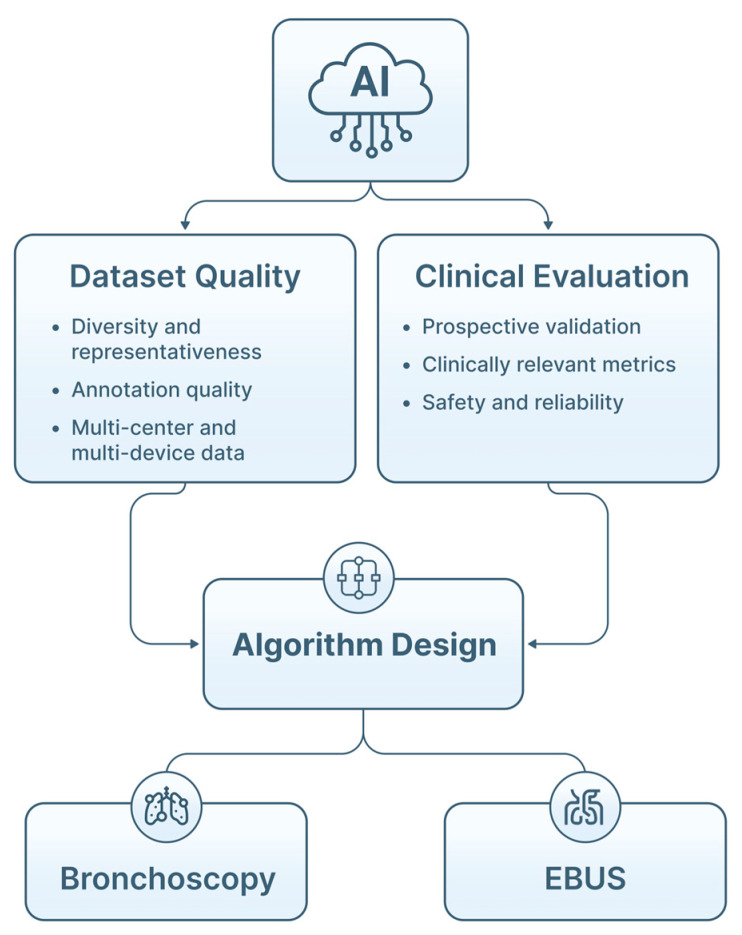
Methodological foundations. Clinical translation relies on continuous interaction between technical development and clinical practice, within a human-in-the-loop framework.

Translation of AI into pulmonary endoscopy depends not only on algorithm performance, but also on dataset quality, methodological rigor, and appropriate clinical evaluation. Table 7 summarizes methodological challenges and evaluation considerations for AI in pulmonary endoscopy. Limitations at these levels directly influence generalizability, safety and real-world reliability (Figure 14).

### 4.1. Dataset Standards and Data Challenges

**Figure 15:**
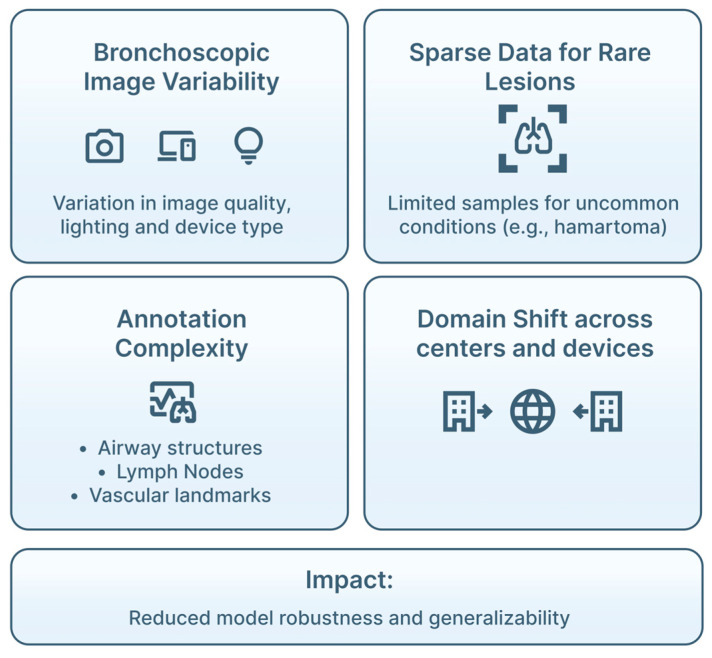
Data challenges in AI pulmonary endoscopy. Bronchoscopic image variability, sparse data for rare lesions, annotation complexity, and domain-shift across centers and devices affect model robustness and generalizability, highlighting the need for large, diverse and well-annotated multi-center datasets.

Endoscopic imaging presents specific constraints for AI development. Bronchoscopic and EBUS data are highly heterogeneous, reflecting differences in anatomy, device settings, operator technique, lighting conditions and motion artifacts. However, AI requires high-quality, representative, and well-annotated data [32,38,39,53,73] (Figure 15). This variability complicates model training and contributes to performance degradation when systems are applied outside the training environment.

High-quality annotation is essential, but challenging. Airway structures, mucosal abnormalities and LN often require frame-by-frame expert delineation, and interobserver variability remains substantial, particularly in EBUS and elastography interpretation [16,32,39,73,74]. In addition, bronchoscopic datasets consist of long video streams with many non-diagnostic frames, increasing the risk that models overfit common patterns while underrepresenting clinically relevant findings [39,52,75]. The difficulty of obtaining consistent annotations limits dataset size and quality, complicating training of segmentation and classification networks.

Patient diversity (e.g., age, sex, smoking history, comorbid lung disease, disease prevalence) must be represented in order to avoid population-specific bias [76]. However, most published datasets remain single-center and device-specific, making domain shift across centers, bronchoscopic platforms and ultrasound systems, a major threat to model robustness [32,38,77]. Approaches such as data augmentation, domain adaptation, fine-tuning and federated learning are being explored, but rigorous external validation remains essential [37,73,78,79,80,81]. Table 8 highlights AI strategies to domain shift mitigation in pulmonary endoscopy.

Sparse data for rare lesions (e.g., early airway dysplasia, uncommon endobronchial tumors, rare LN pathologies) further limits model performance: rare lesions are underrepresented, increasing the risk of misclassification [1,81,82]. Whereas synthetic data and transfer learning offer partial mitigation, real-world diversity is difficult to replicate [19,24,25,83,84].

### 4.2. Algorithm Development and Evaluation

#### 4.2.1. Training, Validation, and Test Splits

Proper dataset partitioning is fundamental to avoid information leakage and overestimation of model performance. Frame-level splits may inadvertently place frames from the same patient or procedure into both training and test sets, artificially inflating accuracy [16,52]. Patient- or procedure-level splits are recommended, ensuring complete separation between training, validation, and test cohorts. External validation on independent datasets is particularly important for bronchoscopy and EBUS AI models, yet remains uncommon in the literature [16,32]. Without such validation, reported performance metrics may not reflect real-world deployment conditions. Table 9 describes the split type and external validation of each published study mentioned in Table 5.

#### 4.2.2. Metrics Appropriate for Bronchoscopy

**Figure 16:**
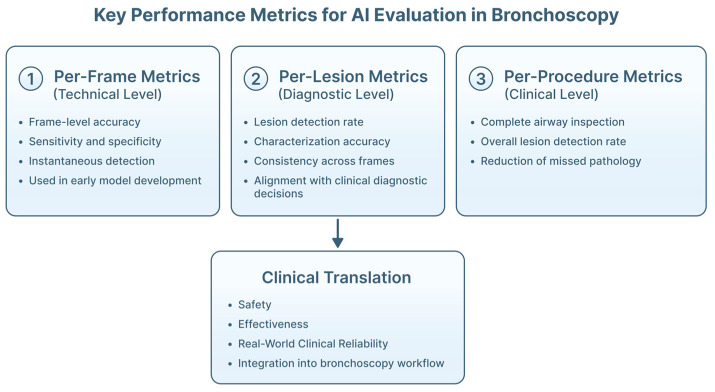
Performance metrics required for the evaluation and deployment of AI algorithms in bronchoscopy. Metrics are organized across different levels of clinical relevance: per-frame metrics assess instantaneous algorithm performance on individual bronchoscopic images; per-lesion metrics evaluate the ability of AI systems to correctly detect or characterize endobronchial abnormalities across multiple frames, aligning more closely with diagnostic decision-making; and per-procedure metrics capture clinically meaningful outcomes. Together, these complementary evaluation layers support robust assessment of AI systems, ensuring that algorithmic performance translates from technical accuracy to real-world clinical integration.

Evaluation metrics should reflect clinical objectives. Per-frame accuracy, frequently reported, has limited clinical relevance in video-based procedures [52]. Per-lesion and per-procedure metrics, such as detection of a lesion during inspection or reduction in missed pathology, better capture real-world utility [85] (Figure 16). For CADe systems, false-positive burden and time to detection are important. For CADx models, discrimination, calibration and predictive value are central [86].

### 4.3. Clinical Trial Design

#### 4.3.1. Observational vs. Interventional Designs

Most AI studies in pulmonary endoscopy are retrospective and observational, assessing model performance on historical datasets [32]. Such designs demonstrate feasibility, but cannot establish clinical benefit. Prospective interventional studies, including randomized controlled trials (RCTs) comparing AI-assisted with standard procedures, are required to determine the effects on diagnostic yield, safety and workflow [23,37] (Table 10).

#### 4.3.2. Real-Time vs. Offline Evaluation

Offline evaluation of curated datasets often overestimates performance relative to real-time deployment, where motion, suboptimal views and operator interaction influence outcomes [23,85]. Real-time studies are therefore necessary to assess latency, usability and human–AI interaction under clinical conditions (Table 11).

#### 4.3.3. Bias, Generalizability, and Reproducibility

Bias may arise from unbalanced datasets, subjective annotation, and selective reporting. Transparent reporting of dataset composition, annotation methods, and subgroup performance is essential [76,79].

Generalizability remains a major challenge, particularly across devices and centers. Reproducibility is also limited by restricted code sharing and inconsistent reporting [23]. Adoption of structured reporting frameworks and multi-center validation are needed to strengthen methodological rigor [86].

## 5. Safety, Ethics, and Regulatory Landscape

Clinical use of AI in pulmonary endoscopy raises important safety, ethical, and regulatory considerations. Unlike offline diagnostic tools, AI systems used during bronchoscopy and EBUS may influence real-time decisions in anatomically constrained and high-risk settings. Responsible deployment therefore requires preservation of clinician oversight, robust risk mitigation and compliance with evolving regulatory frameworks.

### 5.1. Human-in-the-Loop vs. Autonomous Systems

Most AI applications in pulmonary endoscopy are designed as decision-support tools within a human-in-the-loop model, where the bronchoscopist retains full responsibility for procedural decisions. This paradigm is consistent with safety principles in high-risk environments, where contextual judgment and situational awareness remain essential [87,88]. It also aligns with current regulatory expectations, which emphazise clinician oversight, particularly for adaptive or continuously learning systems.

Fully autonomous systems capable of independent navigation or biopsy selection remain largely experimental. Concerns include error propagation, unpredictable edge cases, and limited ability to account for patient-specific factors [89,90]. Evidence from other medical domains suggests that AI performs best as an adjunct rather than a replacement for clinician expertise, particularly in real-time settings [15].

### 5.2. Regulatory Frameworks for AI-Based Medical Devices

AI systems are generally classified as software as a medical device (SaMD). Regulatory agencies are adopting lifecycle-based approaches that emphazise clinical validation, transparency and post-market performance monitoring.

In the United States, the U.S. Food and Drug Administration (FDA) applies a risk-based framework for AI/ML-enabled SaMD, including expectations for real-world performance monitoring and predefined change control plans for adaptive systems [90,91]. In Europe, regulation is governed through the Medical Device Regulation (MDR) and European Medicines Agency (EMA), with requirements for risk classification, data governance and human oversight [92,93]. Real-time decision-support tools for bronchoscopy and EBUS are likely to fall into higher-risk categories, necessitating robust clinical evidence and post-market surveillance. A comparison of key regulatory principles is summarized in Table 12.

### 5.3. Considerations for Real-Time Systems

Real-time AI in the airways poses distinct safety challenges. Errors in navigation or interpretation may lead to missed diagnoses, bleeding, or airway injury [23,38,89]. Systems should therefore incorporate fail-safe mechanisms, strict latency constraints, and confidence estimation to allow clinicians to recognize uncertain outputs. Minimizing false-positive alerts is also important to avoid distraction and procedural inefficiency [38]. Simulation-based testing and phased clinical deployment are recommended to evaluate human–AI interaction before widespread use (Table 13).

### 5.4. Transparency, Explainability, and Medico-Legal Responsibility

Transparency and explainability support clinician trust and safe use of AI-assisted decision support. Clinicians must understand, at least at a conceptual level, how AI systems arrive at recommendations, particularly when those recommendations influence biopsy decisions or staging [94]. Explainable AI (XAI) techniques, such as saliency maps or attention visualization, have been proposed to increase the interpretability of DL models used in endoscopic imaging, but their clinical validity remains under evaluation [95,96]. From a medico-legal perspective, responsibility for clinical decisions currently rests with the physician, even when AI tools are used. This raises important questions regarding liability in cases of AI-related error, particularly if systems are opaque or continuously learning [87]. Clear documentation of AI involvement, system limitations, and clinician oversight is therefore essential.

Professional societies and regulators emphazise that AI should augment rather than obscure clinical reasoning. Transparent reporting, auditability and explicit delineation of responsibility are key ethical requirements for integration into pulmonary endoscopy [95,96].

## 6. Current Evidence and Gaps

### 6.1. Summary of Published AI Tools and Evidence Maturity

Most AI applications in pulmonary endoscopy remain at an early stage of clinical maturity. The majority of studies focus on algorithm development, technical feasibility and retrospective validation, rather than demonstrated clinical impact. Areas with comparatively more evidence include detection of endobronchial lesions, LN classification using EBUS, and navigation support for peripheral pulmonary lesions [18,32,52].

CADe and CADx systems have shown promising diagnostic performance in retrospective datasets, often approaching expert-level accuracy [18,32]. AI-based airway segmentation and VB are also technically advanced and increasingly incorporated into navigation platforms [60]. However, most systems have been evaluated offline, with limited prospective or real-time validation.

Few AI tools have progressed beyond single-center proof-of-concept studies, and comparative interventional trials are rare. Consequently, the evidence base remains heterogeneous and insufficient to support routine guideline endorsement. Table 5 and Table 9 summarize representative AI applications in pulmonary endoscopy and their current evidence maturity.

### 6.2. Methodological Limitations in Current Trials

Common limitations include small sample sizes, particularly for rare lesions, and early mucosal disease, increasing the risk of overfitting and inflated performance estimates [79]. External validation across centers and devices is uncommon.

Prospective interventional evidence is notably lacking. While RCTs have demonstrated the benefits of AI in gastrointestinal endoscopy, comparable trials in bronchoscopy and EBUS are scarce [37]. It therefore remains uncertain whether AI assistance improves diagnostic yield, procedural safety or patient outcomes.

Limited multimodality integration is another gap. Many models rely on a single data source, despite the multimodal nature of pulmonary diagnostics. Integration of radiologic, endoscopic, and pathological data remains underexplored in this field [53].

### 6.3. Barriers to Clinical Adoption

**Figure 17:**
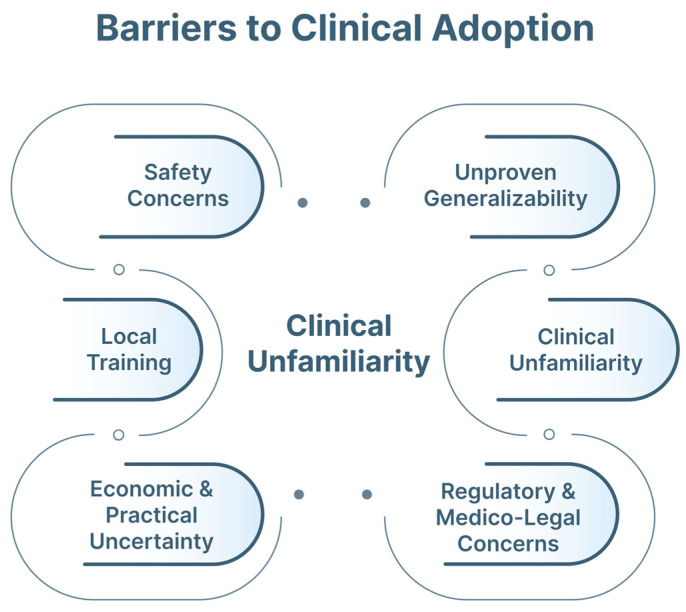
Barriers to clinical adoption of AI in pulmonary endoscopy. Economic constraints, workflow integration challenges, device heterogeneity, regulatory and medico-legal considerations, limited external validation and user training requirements collectively influence successful clinical translation.

Beyond evidentiary gaps, practical factors limit translation (Figure 17). Economic and infrastructure requirements, particularly for advanced navigation or robotic systems, may restrict implementation to high-resource centers [63]. Workflow integration is critical: systems that disrupt procedural flow or increase cognitive load are unlikely to be adopted [97].

**Figure 18:**
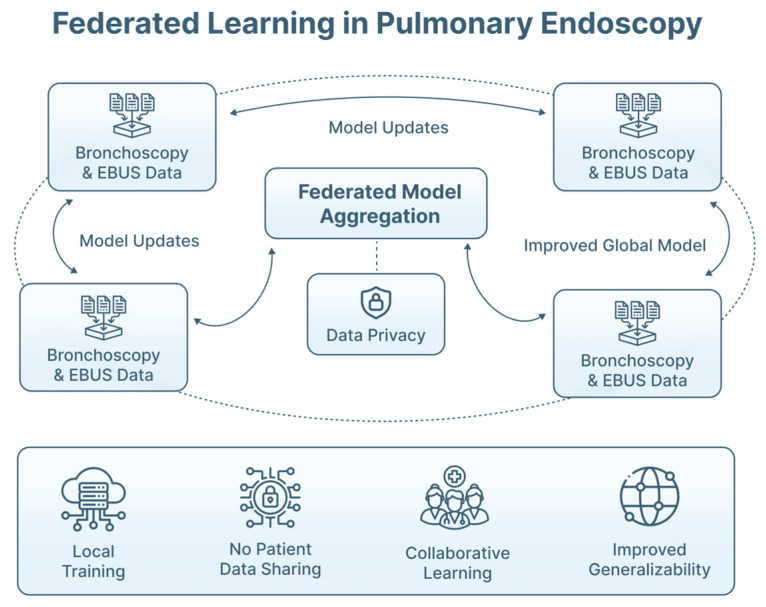
Conceptual overview of federated learning in pulmonary endoscopy. Institutions train AI models locally on bronchoscopic and EBUS data while sharing model updates rather than patient data, supporting collaborative learning, improved generalizability, and preservation of data governance.

Device heterogeneity represents an additional barrier. Variability in bronchoscopes, ultrasound systems, and imaging pipelines contributes to domain shift, reducing performance across institutions [79]. Device-agnostic models and robust external validation are therefore essential. Figure 18 highlights the principal barriers for clinical adoption.

## 7. Future Directions

### 7.1. Robotics and AI Integration for Autonomous Navigation

RB platforms currently operate within a human-in-the-loop framework, providing improved stability, reach, and navigation support, while clinicians retain procedural control [45]. Although these systems enhance access to peripheral lesions, true autonomous navigation is not yet part of routine clinical practice.

Research in simulation and experimental settings has explored AI-enabled semi-autonomous navigation using DL and reinforcement learning (RL) approaches to model airway anatomy and optimize scope trajectories [98,99]. These studies demonstrate the technical feasibility of AI “co-pilot” systems that assist navigation, but clinical translation is constrained by anatomical variability, respiratory motion, CT-to-body divergence and real-time safety requirements [99,100,101].

Near-term progress is therefore more likely through AI-augmented robotic assistance rather than full autonomy [63,100,101]. In such hybrid models, AI may optimize pathways, compensate for anatomical deformation and provide real-time feedback, while the bronchoscopist maintains final authority. This approach aligns with current safety expectations and regulatory frameworks.

### 7.2. Multi-Omic Predictive Models Linked to Endoscopic Findings

Future AI systems are likely to combine endoscopic imaging with radiologic, pathologic and molecular data. Integration of radiomics, digital pathology and genomic or transcriptomic information may support more refined risk stratification and personalized decision-making, particularly in LC [28,53]. Tissue obtained via advanced sampling techniques, such as cryobiopsy, may facilitate these computational approaches.

### 7.3. Federated Learning and Global Data Collaboration

Global collaborations will be essential to address data heterogeneity and the rarity of certain pathologies. Federated learning approaches, in which institutions train models locally while sharing model parameters rather than patient data, offer a potential strategy to improve generalizability, while preserving data governance [77] (Figure 18). Such approaches may support multi-center evidence generation in pulmonary endoscopy.

### 7.4. AI for Training and Quality Improvement

AI-based video analysis may support training by providing objective assessment of airway coverage, procedural completeness, and technical performance [44]. Real-time feedback systems could contribute to more standardized competency assessment and continuous quality improvement, although validation in educational settings is required.

### 7.5. Remote Support and Telementoring

AI-enabled platforms may also facilitate remote supervision and telementoring for complex procedures. By combining real-time video analysis, performance metrics, and decision-support overlays, such systems could help extend expert guidance across centers, potentially improving access to advanced interventional pulmonology expertise [14].

## 8. Conclusions and Discussion

AI is increasingly being applied in pulmonary endoscopy, with developments in image enhancement, lesion detection, navigation, staging, and procedural support. These approaches have the potential to improve diagnostic consistency, reduce operator variability, and support training in complex bronchoscopic and EBUS procedures. In particular, AI-assisted bronchoscopy and EBUS may help address challenges related to subtle mucosal disease, peripheral lesion targeting, and variability in mediastinal staging.

However, the current evidence base remains limited. Most studies are retrospective, single-center, and focused on technical performance rather than clinical outcomes. Reported performance metrics in AI studies, such as accuracy and AUC, do not fully reflect real-world clinical utility. These metrics are typically derived from retrospective, curated datasets and may not capture the complexity of live procedural environments. Clinically meaningful evaluation should extend beyond aggregate performance in order to include per-procedure and per-lesion metrics, false positives per procedure, and time-to-detection, all of which directly influence workflow efficiency and decision-making during pulmonary endoscopy. Furthermore, the impact of AI systems on biopsy targeting, diagnostic yield, and operator behavior remains insufficiently assessed. As such, high algorithmic performance does not necessarily translate into improved clinical outcomes, underscoring the need for prospective, real-time studies that evaluate both effectiveness and integration into routine practice.

Data partitioning strategies and validation approaches vary considerably across published studies and are frequently insufficiently reported. Most studies relied on internal validation, often without clearly specifying whether data splits were performed at the patient, lesion, or frame level. In many cases, image- or frame-level partitioning is likely, which introduces a significant risk of data leakage, which may artificially inflate model performance. External validation was notably absent in nearly all primary studies, limiting the assessment of generalizability across different clinical settings and imaging platforms. These methodological limitations highlight the need for standardized reporting of data partitioning strategies and emphasize the importance of patient-level splits and independent external validation to ensure robust and clinically translatable AI systems in pulmonary endoscopy.

Dataset heterogeneity, domain shift across devices and institutions, annotation complexity, and limited multimodal datasets constrain generalizability. Real-time use in the airway environment introduces additional safety, ethical and regulatory considerations, reinforcing the importance of human-in-the-loop designs, transparent reporting and structured risk mitigation.

Future progress requires a shift from feasibility-driven research toward rigorous clinical validation and responsible implementation. Prospective multi-center studies, including interventional and pragmatic designs, are needed to determine whether AI assistance improves diagnostic yield, safety and workflow. Standardized dataset practices, clinically meaningful evaluation metrics, and post-deployment monitoring should form part of AI development pathways. Alignment with regulatory frameworks for SaMDs and attention to explainability and auditability will be essential to maintain clinician trust and medico-legal clarity.

Successful integration of AI into pulmonary endoscopy will depend on multidisciplinary collaboration between clinicians, data scientists, engineers, regulators and industry partners. Such collaboration will be necessary to ensure that AI systems are clinically relevant, safe and generalizable. With careful validation and governance, AI may become a supportive component of pulmonary endoscopic practice, rather than a standalone technological solution.

## Figures and Tables

**Table 1:** Definitions and clinical applications of pulmonary endoscopic techniques. EBUS-TBNA—endobronchial ultrasound transbronchial needle aspiration; CP-EBUS—convex probe endobronchial ultrasound; CT—computed tomography.

Technique	Definition	Primary Clinical Applications
Flexible Bronchoscopy (White-Light Bronchoscopy, WLB)	Endoscopic visualization of the tracheobronchial tree using a flexible bronchoscope under white-light illumination.	Airway inspection, biopsies, bronchoalveolar lavage, tumor visualization, infection and bleeding assessment.
Autofluorescence Bronchoscopy (AFB)	Bronchoscopy using tissue autofluorescence to enhance contrast between normal and dysplastic mucosa.	Detection of premalignant lesions and early central airway cancer.
Narrow-Band Imaging (NBI)	Optical image enhancement using narrow-band blue and green light to highlight mucosal microvasculature.	Characterization of airway neoplasms, assessment of vascular patterns.
Digital Image Enhancement (i-scan/FICE/SPIES)	Software-based post-processing that enhances surface texture and contrast in real time.	Improved visualization of subtle mucosal abnormalities.
Radial Endobronchial Ultrasound (rEBUS)	Miniature radial ultrasound probe passed through the bronchoscope to image peripheral lung lesions.	Localization and sampling of peripheral pulmonary nodules.
Convex Probe Endobronchial Ultrasound (CP-EBUS)	Bronchoscope with integrated linear ultrasound transducer for real-time needle guidance.	Mediastinal and hilar lymph-node staging, EBUS-TBNA.
EBUS Elastography	Ultrasound-based assessment of tissue stiffness during CP-EBUS.	Differentiation of benign vs. malignant lymph nodes.
EBUS-Guided Transbronchial Needle Aspiration (EBUS-TBNA)	Real-time ultrasound-guided needle sampling of lymph nodes or masses.	Lung cancer staging, diagnosis of mediastinal disease.
Transbronchial Lung Cryobiopsy (TBLC)	Bronchoscopic lung biopsy using a cryoprobe to obtain large, well-preserved tissue samples.	Diagnosis of interstitial lung disease and selected lung tumors.
EBUS-Guided Mediastinal Cryobiopsy	Cryobiopsy of mediastinal lymph nodes under EBUS guidance.	Improved tissue yield for lymphoma diagnosis and molecular testing.
Electromagnetic Navigation Bronchoscopy (ENB)	Navigation system using electromagnetic tracking and CT-based virtual bronchoscopy.	Sampling of small or difficult-to-reach peripheral lesions.
Virtual Bronchoscopy (VB)	CT-derived 3D reconstruction of the bronchial tree for planning and navigation.	Pre-procedural planning and guidance.
Robotic Bronchoscopy (RB)	Robotic-assisted bronchoscopic platforms with articulated catheters and navigation systems.	Precise access to peripheral pulmonary lesions.
Rigid Bronchoscopy	Bronchoscopy performed with a rigid scope under general anesthesia.	Airway obstruction management, tumor debulking, stenting.
Interventional Bronchoscopy (Therapeutic)	Advanced bronchoscopic procedures for airway and pleural disease.	Tumor ablation, stent placement, management of stenosis or hemoptysis.

**Table 2:** Comparison of machine learning (ML) and deep learning (DL) architectures in pulmonary endoscopy. CNNs—convolutional neural networks; CP-EBUS—convex probe endobronchial ultrasound; CT—computed tomography; LSTM—long short-term memory network; rEBUS—radial endobronchial ultrasound; ResNet—residual network; VGG—visual geometry group network; WLB—white-light bronchoscopy.

*Approach*	Typical Algorithms/Architectures	Data Type Best Suited	Strengths in Pulmonology Endoscopy	Limitations
***Traditional Machine Learning (ML)***	Support Vector Machines, Random Forests, Gradient Boosting	Structured data, handcrafted features	Effective for clinical variables, elastography metrics, radiomic features; lower data requirements	Limited performance on raw images; requires manual feature engineering
***Convolutional Neural Networks (CNNs)***	2D CNNs (ResNet, VGG, EfficientNet)	Still images (WLB, rEBUS, EBUS frames)	High accuracy in lesion detection, lymph-node segmentation, rEBUS classification; automatic feature learning	Frame-based analysis may miss temporal context
***Transformer-Based Models***	Vision Transformers (ViT), CNN–Transformer hybrids	Images + multimodal data	Capture global context and long-range dependencies; strong performance in CT and multimodal fusion	Computationally intensive; require large datasets
***3D/Spatiotemporal Deep Learning***	3D CNNs, CNN–LSTM, Video Transformers	Video sequences (bronchoscopy, CP-EBUS)	Incorporate motion, probe dynamics, respiratory variation; improve robustness in real-time procedures	Higher computational cost; complex annotation requirements

**Table 3:** Integrated overview of AI-assisted predictive and prognostic models relevant to pulmonary endoscopy, combining malignancy detection, TNM staging support, and outcome prediction. These models leverage endoscopic imaging, ultrasound, radiologic, pathologic, and multi-omic data through deep learning and multimodal AI approaches. CNN—convolutional neural network; CP-EBUS—convex probe endobronchial ultrasound; CT—computed tomography; DL—deep learning; ML—machine learning; NBI—narrow-band imaging; PET-CT—positron emission tomography; rEBUS—radial endobronchial ultrasound; SUV—standardized uptake value; TBNA—transbronchial needle aspiration.

AI Model Category	Primary Data Inputs	Key AI Techniques	TNM/Clinical Dimension Supported	Predictive/Prognostic Outputs	Clinical Utility in Pulmonary Endoscopy	Evidence Maturity
**Endobronchial Image-Based Models**	White-light bronchoscopy; NBI; autofluorescence; i-scan	CNNs (ResNet, EfficientNet), attention mechanisms	T descriptor (mucosal extent, local invasion)	Probability of malignancy; lesion class; invasion risk	Early airway cancer detection; selection for endobronchial therapy; reduces interobserver variability	Retrospective, mostly single-center
**Radial EBUS-Based Models (rEBUS)**	Radial EBUS grayscale patterns	CNNs; texture-based DL	T descriptor (peripheral lesion characterization)	Malignancy likelihood of peripheral nodules	Improves lesion localization, targeting, and biopsy decision-making	Retrospective validation
**CP-EBUS Multimodal Models**	B-mode EBUS; Doppler; elastography	CNNs + multimodal feature fusion	N descriptor (nodal involvement)	Lymph-node malignancy probability	Enhances mediastinal staging accuracy and lymph-node selection for TBNA	Retrospective clinical validation
**Radiologic CT-Only Models**	Chest CT (nodule morphology, radiomics)	2D/3D CNNs; radiomics + ML	T descriptor (tumor morphology)	Cancer risk score	Pre-procedural risk stratification and target prioritization before bronchoscopy	Retrospective + limited prospective
**PET-CT-Integrated AI Models**	PET uptake (SUV) + CT morphology ± EBUS features	ML/DL fusion models	N and M descriptors (nodal and metastatic risk)	Malignancy probability; metabolic nodal risk	Improves discrimination of benign vs. malignant lymph nodes and staging confidence	Retrospective
**Endoscopy–Radiology Fusion Models**	Bronchoscopic images + CT morphology/radiomics	Multimodal DL; attention-based fusion	T and N descriptors	Integrated malignancy risk; local staging	Reduces CT-to-body divergence; improves targeting and diagnostic yield (especially peripheral lesions)	Early-stage feasibility
**Endoscopy–Pathology AI Models**	Endoscopic findings + digital pathology (biopsy/cryobiopsy)	CNNs on histology + clinical fusion	T and N descriptors (histologic confirmation)	Tumor subtype; invasion depth; diagnostic confirmation	Supports precision diagnosis, molecular testing workflows, and definitive classification	Retrospective
**Multimodal Prognostic/Multi-omic Models**	Endoscopy + CT/PET + genomics/transcriptomics	Deep multimodal networks	Stage grouping (I–IV); overall prognosis	Survival prediction; recurrence risk; therapy response	Enables precision prognostic assessment, treatment planning, and post-procedural surveillance	Experimental/preclinical

**Table 4:** Endobronchial therapies and mechanisms of action.

*Therapy*	Mechanism of Action	Primary Indications	Depth of Effect	Key Advantages	Main Limitations/Risks
***Photodynamic Therapy (PDT)***	Photosensitizing agent activated by specific light wavelength induces cytotoxic reactive oxygen species	Early central airway malignancy, superficial tumors	Superficial (few mm)	High selectivity; preserves airway structure; repeatable	Photosensitivity reactions; delayed effect; limited depth
***Electrocautery***	Direct thermal tissue destruction via electric current	Malignant or benign airway obstruction; tumor debulking	Superficial–moderate	Widely available; immediate effect; low cost	Risk of bleeding, airway fire (oxygen); limited precision
***Argon Plasma Coagulation (APC)***	Non-contact ionized argon gas conducts electrical energy for coagulation	Hemostasis; superficial tumor ablation; granulation tissue	Superficial	Uniform coagulation; reduced contact bleeding	Limited penetration; gas embolism (rare)
***Laser Therapy (*** **e.g.*, Nd:YAG)***	High-energy laser causes photothermal tissue vaporization	Obstructing central airway tumors	Deep (controlled)	Rapid debulking; precise targeting	Expensive; airway fire risk; requires expertise
***Endobronchial Brachytherapy***	Localized radiation delivered via endobronchial catheter	Palliative treatment; recurrent or residual airway tumors	Variable (dose-dependent)	Targeted radiation; spares surrounding tissue	Delayed complications; radiation bronchitis/fistula

**Table 5:** Published studies according to modality, task (CADe/CADx), dataset characteristics, validation strategy, and evidence maturity. Studies are stratified into clinical and technical domains, highlighting sample size, class imbalance (when reported), and validation type, providing context for interpretation of performance metrics and evidence maturity. The table emphasizes the predominance of single-center, retrospective studies with limited external validation, underscoring current barriers to clinical translation. AUC-ROC—area under the curve–receiver operating course; CNN—convolutional neural network; CP-EBUS—convex probe endobronchial ultrasound; LC—lung cancer; LNs—lymph nodes; N—normal; TB—tuberculosis; TTA—test-time augmentation.

Study (Year)	Modality	Task (CADe/CADx)	N (Patients/Lesions/Frames)	Class Imbalance	Validation Type	Study Design	Model/Approach	Main Outcome	Evidence Maturity
**Tan et al. (2018) [40]**	Bronchoscopy images	CADx (lung diseases classification: normal, cancer, TB)	Dataset: 81 (N), 76 (TB), 277 (LC) Not clearly reported	Not reported	Internal split	Retrospective, single-center (preprint)	DenseNet (transfer learning with sequential fine-tuning)	Overall accuracy 77% Detection accuracy: LC (87%), TB (54%), N (91%)	Technical feasibility
**Hotta et al. (2022) [17]**	EBUS	CADx (benign and malignant LC lesions)	213 patients/2.4 M images	Not reported	Internal validation	Retrospective, single-center	CNN	Overall accuracy 83.4%	Observational cohort study
**Li et al. (2021) [41]**	CP-EBUS multimodal	CADe + CADx (intrathoracic LN diagnosis vs. experts)	267 patients/294 LN	Likely imbalanced	Internal validation	Retrospective, single-center	DL multimodal	Overall accuracy 88.57% AUC = 0.9547	Clinical validation
**Zhi et al. (2021) [42]**	EBUS elastography	CADe-enabling (frame selection)	415 LN (trainning/validation); 91 LN (test set)	Not reported	Internal split	Retrospective, single-center	ML selection model	Improved frame selection for diagnosis	Technical feasibility
**Li et al. (2022) [43]**	Bronchoscopy	CADe (lumen recognition)	28,441 images	Not reported	Train/test split	Retrospective, single-center, observational	CNN	Improved procedural quality	Retrospective technical validation
**Yoo et al. (2021) [44]**	Bronchoscopy video	CADe (anatomical recognition)	Not explicitly stated (video frames) 3216 patients; 47,447 images	Not reported	Internal validation	Retrospective, single-center, offline test	CNN (with temporal smoothing)	High accuracy airway recognition (86%)	Technical validation
**Banach et al. (2021) [45]**	Bronchoscopy + CT	Navigation (CADe-support)	Not clinical dataset (simulation + paired data)	Not applicable	Technical validation	Preclinical/simulation-based	CycleGAN-based DL	Improved CT-video registration	Preclinical/technical feasibility
**Deng et al. (2022) [46]**	White-light bronchoscopy	CADe/CADx	2238 lesions (666 malignant/152 benign)	Imbalanced (~81% malignant)	Internal test set	Retrospective, single-center	ResNet DL	High diagnostic performance, compared with clinicians Overall accuracy = 0.951 AUC = 0.940	Retrospective clinical validation
**Yu et al. (2023) [47]**	rEBUS	CADx (benign vs. malignant)	Retrospective image-level dataset (769 + 615 + 154 + 300) Frame/image level annotation	Not reported	Internal validation (with TTA)	Retrospective, multi-center	CNN + TTA	High accuracy AUROC = 0.88	Clinical validation
**Vu et al. (2024) [48]**	Bronchoscopy video	CADe (dataset + segmentation)	106 LC patients, 102 non-LC patients	Balanced dataset	Benchmark split	Retrospective dataset single-center study	UNet++/ESFPNet	Dataset + baseline performance	Preclinical dataset
**Hu et al. (2024) [49]**	EBUS	CADx, benign vs. malignant LNs (radiomics)	197 patients (LN-based dataset)	Not reported	Internal validation	Retrospective, single-center	Radiomics + ML	Training group: AUROC 0.892; accuracy 85.3% Validation group: AUROC 0.906; accuracy 74.2%	Clinical validation No real-time clinical impact assessment
**Ervik et al. (2024) [16]**	EBUS	CADe (segmentation of LNs)	56 patients/28,134 images	Not reported	Train/val/test split	Retrospective	U-Net DL	Real-time segmentation; overall accuracy = 59.5%	Technical + real-time feasibility
**Liu et al. (2025) [50]**	Bronchoscopy	CADe (tumor detection)	Not clearly reported (self-built tumor dataset)	Not reported	Internal validation	Retrospective, single-center, exploratory	KD-MFAD (unsupervised DL)	AUC 97.6% Improved 5–10%% of baseline performance	Exploratory feasibility
**Tang et al. (2025) [18]**	EBUS (meta-analysis)	CADx	6090 LNs (12 studies)	Variable across studies	Pooled analysis	Systematic/meta-analysis	DL models	Strong pooled diagnostic performance (benign vs. malignant) AUROC = 0.9	Retrospective validation

**Table 6:** Virtual broncoscopy, AI-guided virtual bronchoscopy and robotic bronchoscopy. AI-guided navigation systems offer improved airway segmentation, pathway optimization, and potential real-time adaptability, at the expense of increased technical complexity and currently limited high-level clinical evidence. They represent a continuum of increasing technical sophistication, with corresponding gains in navigation precision and procedural stability, but also rising complexity and cost.

	Conventional Virtual Bronchoscopy (VB)	AI-Guided Virtual Bronchoscopy Navigation	Robotic Bronchoscopy (RB)
DEFINITION	CT-based 3D reconstruction of the tracheobronchial tree, used for visualization and pre-procedural planning.	Virtual bronchoscopy augmented with AI, for automated airway segmentation, target identification, and navigation support.	Robotic catheter-based bronchoscopy systems with integrated navigation, stability control, and articulated reach.
AIRWAY SEGMENTATION	Rule-based or semi-automatic; limited in distal or diseased airways.	Deep learning-based segmentation, with improved extraction of subsegmental and peripheral airways.	AI-enhanced segmentation integrated into robotic planning software.
NAVIGATION CAPABILITY	Static, pre-procedural only.	Near-real-time or real-time guidance when integrated with navigation systems.	Real-time navigation with robotic control and continuous positional feedback.
TARGET LOCALIZATION	Operator-dependent interpretation of CT anatomy.	AI-assisted pathway optimization and predicted lesion proximity.	High-precision targeting with catheter stability and fine distal control.
CT-TO-BODY DIVERGENCE HANDLING	No compensation for divergence.	Partial compensation using AI models and dynamic registration.	Advanced compensation via sensor feedback, robotics, and AI-based deformation modeling.
OPERATOR DEPENDENCY	High.	Moderate; AI supports but does not replace operator decisions.	Lower for navigation precision, but still requires expert oversight.
PROCEDURAL STABILITY	Manual scope control; limited stability in distal airways.	Manual control enhanced by AI guidance.	Robotic articulation provides superior distal stability and control.
INTEGRATION WITH RADIAL EBUS/EBUS	Requires manual coordination.	AI can assist in interpreting rEBUS positioning.	Seamless integration with rEBUS and tool-in-lesion confirmation.
IMPACT ON DIAGNOSTIC YIELD	Indirect; improves planning but limited effect alone.	Potential improvement, particularly for small or complex lesions.	Demonstrated improvement in accessing small, peripheral, or bronchus-sign-negative lesions.
SAFETY CONSIDERATIONS	Very low risk; only a planning tool.	Requires fail-safes and human-in-the-loop oversight.	Requires robust safety systems due to robotic actuation.
EVIDENCE MATURITY	Established and widely used.	Emerging; mainly retrospective and early prospective studies.	Early-to-intermediate; growing prospective and real-world evidence.
COST AND COMPLEXITY	Low cost, minimal infrastructure.	Moderate cost; software and integration requirements.	High cost; specialized equipment, training, and maintenance.

**Table 7:** Methodological challenges, impact on AI systems and solutions for AI in pulmonary endoscopy.

Domain	Challenge/Consideration	Impact on AI Systems	Mitigation/Best Practice
Dataset variability	Heterogeneous imaging conditions (lighting, motion, device settings, operator technique)	Reduced generalizability; performance drop across centers	Diverse, multi-center datasets; data augmentation; external validation
Annotation complexity	Frame-level labeling of airway structures, mucosal lesions, lymph nodes; interobserver variability	Noisy ground truth; inconsistent training signals	Expert consensus annotation; adjudication; interobserver agreement analysis
Video data redundancy	Large numbers of non-diagnostic or repetitive frames	Model bias toward common patterns; underrepresentation of rare findings	AI-assisted or expert-guided representative frame selection
Rare lesion scarcity	Underrepresentation of uncommon airway and nodal pathologies	Poor performance in clinically critical but infrequent cases	Data sharing, synthetic data, transfer learning; cautious interpretation
Domain shift	Differences across centers, devices, ultrasound systems, and workflows	Degradation when deployed outside training environment	Domain adaptation; fine-tuning; federated learning; rigorous external testing
Dataset partitioning	Frame-level splits causing data leakage	Overestimated performance metrics	Patient- or procedure-level splits; strict separation of cohorts
Evaluation metrics	Reliance on per-frame accuracy	Limited clinical relevance	Use per-lesion and per-procedure metrics; assess false positives and calibration
Study design	Predominance of retrospective observational studies	Uncertain clinical impact	Prospective interventional studies; randomized controlled trials
Offline vs. real-time testing	Performance assessed only on curated datasets	Overestimation of real-world utility	Real-time evaluation; assessment of latency, usability, and human–AI interaction
Bias	Unbalanced datasets; subjective labeling; selective reporting	Reduced performance in underrepresented populations	Transparent reporting; bias audits; subgroup analysis
Generalizability	Limited cross-center validation	Failure during real-world deployment	Multi-center validation; domain-robust training
Reproducibility	Limited code/data sharing; inconsistent reporting	Reduced scientific credibility	Standardized reporting frameworks; open protocols where feasible
Workflow mismatch	Trial conditions differ from routine practice	Overestimated benefit	Pragmatic study designs; real-world evaluation

**Table 8:** Strategies to domain shift mitigation in AI applications for bronchoscopy.

*Technique*	*Core Principle*	*Application in Bronchoscopy*	*Key Advantages*	*Limitations/Considerations*
**Data Augmentation**	Artificially increases data diversity by applying transformations to training images	Simulation of variability in lighting, mucus, blood, camera angle, and motion in bronchoscopic videos	Improves robustness to visual variability across procedures and operators	May not fully capture device-specific or center-specific differences
**Domain-Adversarial Training**	Learns feature representations that are invariant across domains using adversarial objectives	Reduces performance degradation when models are deployed across different bronchoscopes or institutions	Enhances cross-center generalizability without requiring labeled target data	Increased training complexity; may be unstable without careful tuning
**Style Transfer**	Transforms images from one domain to match the visual style of another (e.g., via GANs)	Harmonizes appearance between bronchoscopic systems, imaging settings, and centers	Explicitly addresses device- and center-dependent visual differences	Risk of introducing artificial features, if not carefully validated
**Fine-Tuning with Local Data**	Adapts a pretrained model using a small labeled dataset from the target center	Tailors AI systems to local bronchoscopic equipment, workflows, and patient populations	Often yields substantial performance gains with limited additional data	Requires local annotation effort; may reduce generalizability if over-fitted

**Table 9:** Type of data split and external validation of published studies of AI in pulmonary endoscopy. Most studies relied on internal validation strategies, frequently without explicit reporting of the level of data partitioning (patient-, lesion-, or frame-level). Where not specified, image- or frame-level splitting is likely, which may introduce data leakage and lead to overestimation of model performance. External validation was absent in nearly all primary studies, with the exception of indirect evidence from meta-analytic synthesis.

Study (Author, Year)	Type of Data Split	External Validation
Tan et al., 2018 [40]	Internal split (not specified; likely image-level)	No
Hotta et al., 2022 [17]	Internal validation (split not specified; likely image-level)	No
Li et al., 2021 [41]	Internal validation (not specified)	No
Zhi et al., 2021 [42]	Train/validation/test split (level not specified)	No
Li et al., 2022 [43]	Train/test split (image-level)	No
Yoo et al., 2021 [44]	Internal validation (frame/video-level)	No
Banach et al., 2021 [45]	Technical validation (simulation-based; no clinical split)	No
Deng et al., 2022 [46]	Internal test set (lesion/image-level)	No
Yu et al., 2023 [47]	Internal validation (image-level, with test-time augmentation)	No
Vu et al., 2024 [48]	Benchmark split (dataset-level; image/video-based)	No
Hu et al., 2024 [49]	Internal validation (patient/LN-level not explicitly specified)	No
Ervik et al., 2024 [16]	Train/validation/test split (image-level)	No
Liu et al., 2025 [50]	Internal validation (not specified)	No
Tang et al., 2025 [18]	Pooled analysis (meta-analysis; study-level aggregation)	Yes (indirect)

**Table 10:** Observational and interventional studies used to evaluate AI systems in pulmonary endoscopy: differences in clinical risk, outcome measures, and evidentiary strength relevant to bronchoscopy, EBUS, and interventional pulmonology.

*Aspect*	*Observational Designs*	*Interventional Designs*
***Definition***	AI is evaluated without influencing clinical decisions	AI actively influences or supports clinical decision-making
***Typical Study Types***	Retrospective cohorts, prospective observational studies	Randomized controlled trials (RCTs), pragmatic trials
***Role of AI***	Silent or “shadow mode” evaluation	Decision support or real-time guidance
***Clinical Risk***	Minimal (no impact on patient care)	Higher (AI may affect diagnosis or intervention)
***Primary Outcomes***	Accuracy, sensitivity, specificity, agreement with experts	Diagnostic yield, complication rates, procedure time, patient outcomes
***Strengths***	Safer early validation, easier regulatory approval	Direct assessment of clinical utility and benefit
***Limitations***	Limited evidence of real-world impact	Higher cost, ethical and regulatory complexity
***Typical Use Case***	*Early-stage validation of CADe/CADx algorithms*	*Demonstration of clinical benefit and workflow integration*

**Table 11:** Offline versus real-time evaluation paradigms for AI systems in pulmonary endoscopy: differences in latency requirements, safety considerations, regulatory scrutiny, and clinical relevance for AI deployment in bronchoscopy and EBUS. SaMD—software as a medical device.

*Aspect*	*Offline Evaluation*	*Real-Time Evaluation*
***Timing of AI Output***	Post-procedure or retrospective analysis	During live bronchoscopy or EBUS
***Clinical Interaction***	No interaction with operator	Continuous interaction with bronchoscopist
***Latency Requirements***	Not time-critical	Strict latency constraints
***Typical Metrics***	Per-frame or per-lesion accuracy	Per-procedure outcomes, missed lesions, workflow impact
***Safety Considerations***	Low risk	High importance of fail-safes and confidence thresholds
***Technical Complexity***	Lower	Higher (integration with hardware and software systems)
***Regulatory Scrutiny***	Moderate	High (real-time SaMD requirements)
***Typical Use Case***	Algorithm development and benchmarking	Clinical deployment and decision support

**Table 12:** Regulatory frameworks adopted by the U.S. Food and Drug Administration (FDA) and the European regulatory system for artificial intelligence (AI)-based software as a medical device (SaMD).

*Aspect*	FDA (United States)	EMA/EU Regulatory Framework (Europe)
***Regulatory Basis***	FDA Medical Device Regulations; digital health & SaMD guidance	EU Medical Device Regulation (MDR 2017/745) and proposed EU AI Act
***Definition of SaMD***	Software intended for medical purposes independent of hardware	Software with medical purpose classified as a medical device under MDR
***Risk Classification***	Class I–III based on intended use and risk to patient	Class I, IIa, IIb, III based on clinical risk and decision impact
***AI-Specific Guidance***	FDA AI/ML SaMD action plan; Good Machine Learning Practice (GMLP)	No AI-only pathway yet; AI regulated via MDR + horizontal AI Act
***Human-in-the-Loop Emphasis***	Strong emphasis on clinician oversight for decision-support systems	Human oversight explicitly required under proposed EU AI Act
***Handling of Adaptive/Learning AI***	Predetermined change control plan (PCCP) proposed for adaptive AI	Continuous learning currently restricted; major changes require re-certification
***Clinical Evidence Requirements***	Analytical validation, clinical validation, real-world performance	Clinical evaluation report (CER), performance and safety evidence
***Post-Market Surveillance***	Real-world performance monitoring and post-market studies	Mandatory post-market surveillance and vigilance reporting
***Explainability & Transparency***	Encouraged, especially for high-risk clinical decision support	Explicit transparency and explainability obligations in AI Act
***Relevance to Pulmonology Endoscopy***	Applies to CADe/CADx, navigation and real-time decision support tools	Applies to bronchoscopy, EBUS, and interventional AI systems

**Table 13:** Real-time AI systems in pulmonary endoscopy: risk mitigation strategies.

*Risk Mitigation Strategy*	*Description*	*Clinical Relevance in Pulmonary Endoscopy*
***Fail-safe mechanisms***	System designs that ensure procedures can continue safely if the AI system fails, disconnects, or produces no output.	Prevents procedural interruption during bronchoscopy or EBUS, ensuring patient safety even if AI assistance becomes unavailable.
***Latency constraints***	Strict limits on processing and response times to guarantee that AI outputs are delivered within clinically meaningful timeframes.	Avoids misleading guidance during dynamic airway maneuvers, navigation, or biopsy targeting where delayed feedback may be unsafe or irrelevant.
***Confidence estimation***	Algorithms that quantify prediction uncertainty and signal low-confidence outputs when inputs deviate from the training distribution.	Enables bronchoscopists to recognize unreliable AI recommendations, supporting informed human override and safer decision-making.

## Data Availability

No new data were created or analyzed in this study.
